# Running Reverses Chronic Stress‐Induced Changes in Serotonergic Modulation of Hippocampal Granule Cells and Altered Behavioural Responses

**DOI:** 10.1111/ejn.70084

**Published:** 2025-03-31

**Authors:** Carmen Soto, Lazaro P. Orihuela, Grego Apostol, Carmen Vivar

**Affiliations:** ^1^ Laboratory of Neurogenesis and Neuroplasticity. Department of Physiology, Biophysics and Neuroscience Centro de Investigacion y de Estudios Avanzados del Instituto Politécnico Nacional Mexico City Mexico

**Keywords:** 5‐HT1_A_, 5‐HT_3_, chronic stress, dentate gyrus, hippocampus, running, serotonin

## Abstract

Chronic stress increases susceptibility to anxiety and depression disorders, recurrent and common psychiatric conditions. Current antidepressant medications have varying degrees of efficacy and often have multiple side effects limiting treatment adherence. Physical exercise has beneficial effects on stress‐related mental disorders. However, the underlying mechanisms are unclear. Dentate gyrus granule cells (GCs) excitability may mediate stress resilience. Here, we expose young adult C57Bl6 mice to chronic restraint stress (CRS) for 14 days followed by 30 days of running treatment. Behavioural evaluation before and after treatment showed that the behavioural alterations elicited by CRS were mitigated by running. Next, we evaluated serotonergic modulation of GC excitability, as a potential mechanism underlying running‐induced stress resilience. Electrophysiological recordings indicate that CRS alters serotonergic modulation of GC excitability. Utilising (S)‐WAY 100135 and Tropisetron, antagonists of 5‐HT_1A_ and 5‐HT_3_ receptors respectively, we show that running recovers 5‐HT_1A_ receptor activity lost by CRS. Additionally, running promotes the indirect modulation of GCs through 5‐HT_3_ receptor activation. Thus, 5‐HT_1A_ and 5‐HT_3_ receptors may be potential targets for the treatment of stress‐related psychiatric disorders.

## Graphical abstract

Chronic stress restraint (CRS) induces altered behavioural responses that are mitigated by running treatment. We evaluated serotonergic modulation of hippocampal granule cells (GC) excitability, as a potential mechanism underlying exercise‐induced anxiolytic and antidepressive effects. CRS modifies the serotonergic modulation of GC excitability. Running recovered 5‐HT_1A_ receptor activity lost by CRS, and promoted the indirect modulation of GCs through 5‐HT_3_ receptor activation, suggesting these two receptors as potential targets for the treatment of stress‐related psychiatric disorders.

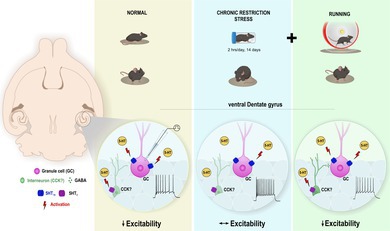

**Abbreviations**
ACSF
: artificial cerebrospinal fluid
AP
: action potential
CCK
: cholecystokinin
CRS
: chronic restraint stress
CON
: control
DG
: dentate gyrus
FST
: forced swimming test
GABA
: γ‐aminobutyric acid
**GABA**_**A**_
: γ‐aminobutyric acid A receptor
GCs
: granule cells
OFT
: open field test
POST
: after exposition
PRE
: before exposition
PTX
: picrotoxin
PV
: parvalbumin
**R**_**in**_
: Input resistance
RMP
: resting membrane potential
RUN
: running
S‐WAY
: (S)‐WAY 100135
SEM
: standard error of the mean
TST
: tail suspension test
TROPI
: Tropisetron
vDG
: ventral dentate gyrus
+CRS
: chronic restraint stress group
5‐HT
: serotonin
**5‐HT**_**1A**_
: serotonin 1A receptor
**5‐HT**_**3**_
: serotonin 3 receptor
−CRS
: control non‐stressed group

## 1 Introduction

Anxiety and depression are the most common mental disorders worldwide (Ferrari et al. 2024). The vulnerability to these disorders increases with exposure to chronic stress, producing alterations at cellular and molecular levels in the hippocampus (Kim and Diamond 2002; McEwen, Nasca and Gray 2016; McEwen and Akil 2020; Tran and Gellner 2023). Specifically, the ventral hippocampus is linked to emotional processing, such as anxiety and stress regulation and among the hippocampal subfields the dentate gyrus (DG) is susceptible to damage by stress hormones (McEwen 1999; Fanselow and Dong 2010; Segal, Richter‐Levin and Maggio 2010). Moreover, serotonin (5‐hydroxytryptamine; 5‐HT) has been implicated in the regulation of anxiety and depression (Żmudzka et al. 2018; Lin et al. 2023). Medications targeting serotonergic signalling, such as fluoxetine, are currently the first‐line treatment for these disorders (Lin et al. 2023). Indeed, deletion of the 5‐HT_1A_ receptor in the granule cells (GCs) of the DG abolishes the effects of fluoxetine (Samuels et al. 2015), suggesting that GCs may contribute to stress resilience.

Despite advancements in the development of therapeutics, current treatment options have not reached optimal efficacy and often have multiple side effects that limit treatment adherence (Unni, Gupta and Sternbach 2024). Therefore, novel treatment strategies are still needed. Studies in humans have shown that physical exercise, with moderate‐to‐high intensities, prevents and ameliorates depression and anxiety (Carek, Laibstain and Carek 2011; Kandola et al. 2019; Singh et al. 2023). However, the underlying mechanisms remain unclear. Studies in rodents have shown that exercise elicits cellular and molecular changes, mainly in the DG of the hippocampus (Vivar, Potter and van Praag 2012; Voss et al. 2013, 2019; Vivar and van Praag 2017; Kandola et al. 2019; Gao, Syed and Zhao 2023), including the increment of 5‐HT levels and the activation of 5‐HT_3_ receptors (Gomez‐Merino et al. 2001; Kondo et al. 2015; Pietrelli et al. 2018). Altogether suggests that DG and specifically the GCs may contribute to exercise‐induced anxiolytic and antidepressant effects. Indeed, chronic stress during social defeat increases the excitability of GCs (Schoenfeld et al. 2013; Anacker et al. 2018), whereas their chemogenetic inhibition confers stress resilience (Anacker et al. 2018). Moreover, 5‐HT_1A_ receptor activation in GCs from ventral DG (vDG) prevents stress‐induced hyperexcitability conferring resilience to chronic stress (Bickle et al. 2024).

To understand the mechanisms underlying exercise‐induced stress resilience, we assessed the exercise‐induced effects on GC excitability. We induced chronic stress through daily restraint treatment that elicited altered behavioural responses (Kim and Han 2006). Chronic restraint stress (CRS) was followed by 30 days of running. Next, we evaluated the role of 5‐HT on the modulation of GC excitability. We show that running recovers CRS‐induced loss of 5‐HT_1A_ receptor activity. Furthermore, our results suggest that 5‐HT regulates GABAergic synaptic transmission through 5‐HT_1A_ and 5‐HT_3_ receptor activation thereby indirectly modulating GC excitability. Thus, 5‐HT_1A_ and 5‐HT_3_ receptors may be targets for the treatment of stress‐related psychiatric disorders.

## 2 Material and Methods

### 2.1 Animals and Housing Conditions

**FIGURE 1:**
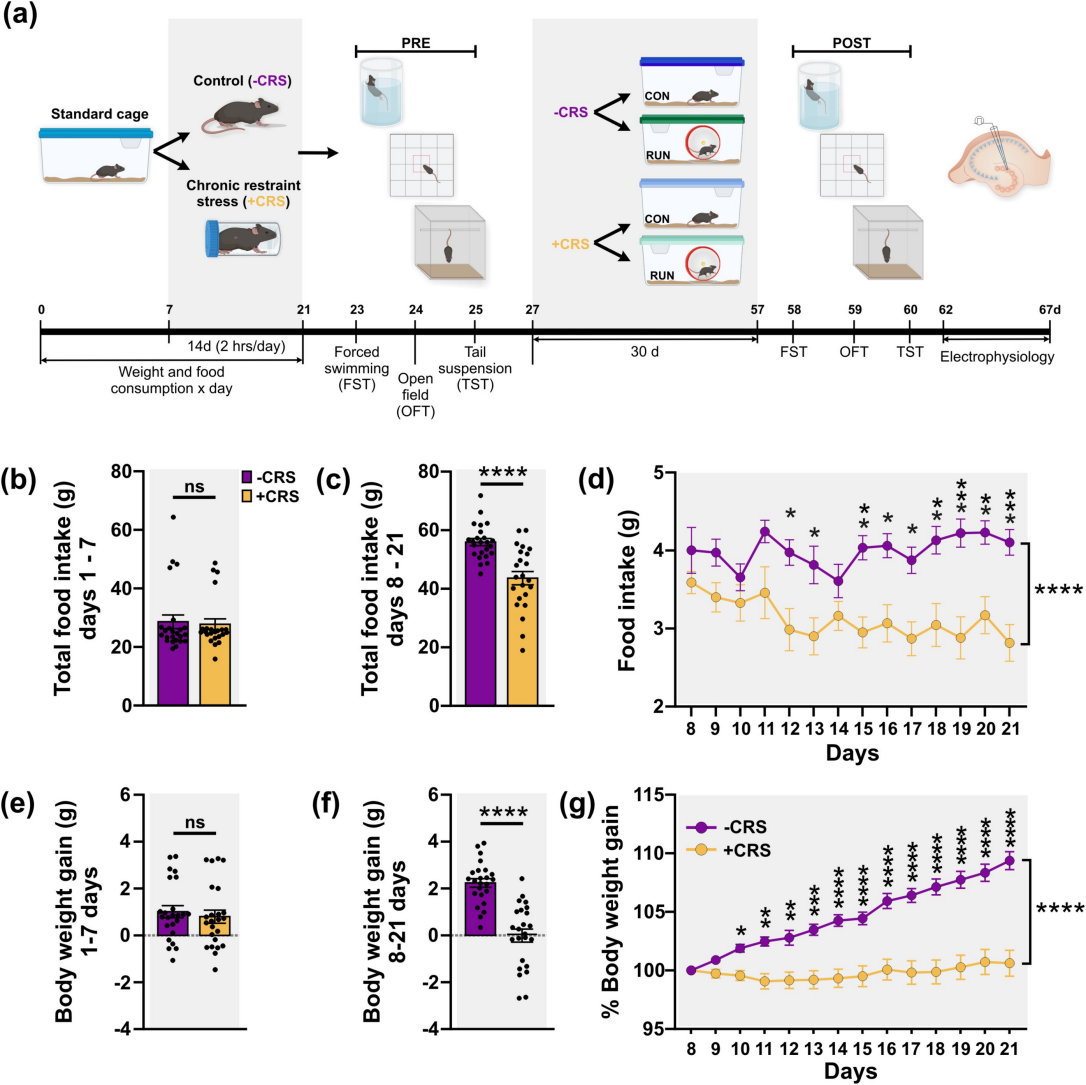
Chronic restraint stress decreases body weight gain and food intake. (a) Timeline of the experiment. Adult young mice were housed in standard conditions for 7 days. Thereafter, mice were randomly assigned to chronic restraint stress (+CRS; *n* = 24) or control non‐stressed (−CRS: *n* = 23) group. Body weight and food intake were monitored daily from day 1 to 21. The CRS was applied 2 h/day for 14 days. Mice from −CRS and +CRS groups were randomly subdivided into two groups and housed under control sedentary or running conditions for 30 days. Avoidance behaviour and coping strategies during acute inescapable stress were assessed before (PRE) and after (POST) the treatment using the forced swimming test (FST), tail suspension test (TST) and open field test (OFT). Thereafter, patch‐clamp recordings were performed on ventral dentate gyrus granule cells (GCs). (b) Before exposure to +CRS or −CRS, total food intake, from days 1–7, was similar between groups. (c) Total food intake, from days 8–21, was significantly reduced in +CRS mice compared to non‐stressed mice. (d) Daily food intake was significantly reduced over time by CRS. (e) Body weight gain was similar between groups before (days 1–7) CRS. (f) Chronic restraint stress exposure reduces body weight gain. (g) Percentage of daily body weight gain during the period of exposure (days 8–21) to CRS or control conditions. Restrained mice weighed less than control mice from Day 3 of CRS until the end of the exposure. Unpaired *t*‐test, 2‐tailed was used for comparison. For (d) and (g)*,* two‐way ANOVA with repeated measures followed by Bonferroni's *post‐hoc* test was used. Values are mean ± SEM. Statistical significances are denoted by *p ≤ 0.05, ** *p* ≤ 0.01, ***p ≤ 0.001, **** *p* ≤ 0.0001, ns *p* > 0.05.

Male C57Bl/6 mice (4 to 6 weeks old) were obtained from the Laboratory Animal Production and Experimentation Unit (UPEAL) of the Center for Research and Advanced Studies (CINVESTAV). Mice were individually housed in Super Mouse 750 cages (483 cm^2^ floor area; Lab Products, LLC), with water (Hydropac water pouch, Lab Products, LLC) and food (PicoLab Mouse Diet 20, 5058, LabDiet. Percentage of energy from protein: 23.217%, carbohydrate: 55.217% and fat: 21.566%; 3.6 kcal/g) *ad libitum*, in a 12 h/12 h light/dark cycle (light on at 6:00 am), with controlled temperature (21°C–23°C) and humidity (50 ± 5%). As standard, the cages included bedding (wood shaving), nesting material (1‐Ply toilet paper) and a cardboard tube (8.5 cm height × 2 cm diameter). Following a 1‐week acclimatization period, mice were randomly assigned to the chronic restraint stress (+CRS) or Control non‐stressed (−CRS) group (Figure 1a). Body weight and food intake were monitored daily. All mice received 200 g of mouse chow each morning. The following day, the remaining food, including the spilled food, was weighed. All manipulations were conducted during the light phase of the day. The voluntary exercise was performed in a silent spinner running wheel (11.5 cm diameter; Kaytee Silent Spinner). Wheels were linked to an odometer (bicycle computer, SD‐548C, SunDing) to measure daily running distance. All procedures were performed according to the Official Mexican Standard for the Management and Laboratory Animal Care (NOM‐062) and approved by UPEAL of the CINVESTAV and in compliance with the NIH laboratory animal care guidelines.

### 2.2 Chronic Restraint Stress

The CRS was applied for 2 h per day (11:00–13:00 h), for 14 days during the light phase (Figure 1a). Mice were placed into a well‐ventilated 50 mL polypropylene conical tube (inner diameter, 28 mm; length, 115 mm), introducing the head of the mice first and finally placing the cap of the tube. Mice weighted ~22–28 g and were not able to move forward or backward in this device. The tubes were horizontally placed inside a clean cage, one per cage. Afterward, mice were returned to their home cage each day. Control mice remained in their cages, in the testing room, without being disturbed (Kim and Han 2006; Jones, Zhou and Jhaveri 2022). For all the mice, food and water were restricted during the 2 h of CRS or control conditions.

### 2.3 Behavioural Test

All the behavioural tests were conducted twice, after either CRS or unstressed conditions (PRE) and after either control sedentary or running exposition (POST) (Figure 1a). The noise in the behaviour testing room was minimal as it was located in an undisturbed area in the vivarium. Mice were habituated to the testing room by placing the home cage in the room 30 min before testing. The behavioural testing was performed between 10:00–13:00 h. Sessions were video recorded using a webcam (Logitech HD Pro Webcam C920, Logitech Europe, S.A) connected to a computer using the free application CameraFi2 (Vault Micro, Inc). The analysis was performed using the Mouse Behavioural Analysis Toolbox (MouBeAT, Bello‐Arroyo et al. 2018) and Kinoscope software (Kokras et al. 2017) which was blinded to animal conditions. All apparatuses were cleaned with 70% ethanol between every mouse.

#### 2.3.1 Forced Swimming Test (FST)

Each mouse was placed into a glass cylindrical container (diameter, 16.8 cm; height, 25 cm), filled with tap water at 25 ± 1°C to a depth of 18 cm. The test session lasted 6 min and was video‐recorded placing the camera in front of the container and analysed by a researcher blind to experimental groups. The duration of immobility behaviour (the time during which the mouse made only the minimal movements necessary to keep their heads above water) was measured during the first 5 min.

#### 2.3.2 Open Field Test (OFT)

The test was performed in a square chamber (40 × 40 × 40 cm; Controladores Electrónicos Digitales). Animals were placed in the centre of OFT and locomotor activity was video recorded for 10 min. The total area of the chamber was divided into 16 squares (10 × 10 cm). The centre was defined as a 20 × 20 cm area in the centre of the open field. The total distance travelled and the time in the centre area were analysed.

#### 2.3.3 Tail Suspension Test (TST)

Animals were suspended from a horizontal bar at a distance of 30 cm from the surface with adhesive tape placed approximately 1 cm from the tip of the tail (Seo et al. 2012; Son et al. 2018). Individual tests lasting 6 min were video‐recorded and analysed. The immobility time was analysed during the first 4 min of the video.

### 2.4 Electrophysiology

One to 5 days after the last behavioural evaluation (POST), mice were anaesthetized with isoflurane and decapitated. Brains were removed into a chilled solution containing (in mM): 110 Choline‐Cl, 2.5 KCl, 2 NaH_2_PO_4_, 25 NaHCO_3_, 20 glucose, 0.5 CaCl_2_, 7 MgCl_2_, 0.6 Na^+^ pyruvate, 1.3 Na^+^ ascorbate and 1 kynurenic acid. Horizontal ventral hippocampal slices (350 μm thick) were obtained (Leica VT1200) and transferred to a chamber containing artificial cerebrospinal fluid (ACSF) in mM: 125 NaCl, 2.5 KCl, 2 NaH_2_PO_4_, 25 NaHCO_3_, 2 CaCl_2_, 1.3 MgCl_2_, 10 glucose, 3.1 Na^+^ pyruvate, 1.3 Na^+^ ascorbate, bubbled with 95% O_2_/5% CO_2_ (pH 7.4, 310 mOsm). Slices were incubated at 30°C for 30 min and stored at room temperature afterward. Electrophysiological recordings were carried out in ACSF at 28 ± 1°C using microelectrodes (3–5 MΩ) pulled from borosilicate glass (G150F‐4, Warner Instruments) containing (in mM): 120 K^+^‐gluconate, 20 KCl, 5 NaCl, 4 MgCl_2_, 0.1 EGTA, 10 HEPES, 4 Tris‐ATP and 10 phosphocreatine (pH 7.4, 295 mOsm). The GCs from the outer portion of the GC layer (GCs‐OGCL) were recorded, which are suggested to be GCs born during development (Nowakowski and Rakic 1981). Whole‐cell patch‐clamp recordings (Multiclamp 700B, Axon Instruments) were filtered at 2 kHz and digitalized at 20 kHz (Digidata 1322A and pClamp 10.2 Software; Molecular Devices). Series resistance was typically 10–30 MΩ, recordings were discarded if access resistance changed by >30% during the recordings. Recordings were not corrected for a liquid junction potential. Data was analysed using Clampfit 10.2 software (Molecular Device). Whole‐cell recordings were performed in the current clamp configuration to analyse the intrinsic properties of the GCs. To measure the R_in_, we used a protocol of hyperpolarizing pulses (100 pA, 3 s of duration, at a frequency of 0.083 Hz). To measure the spiking threshold and reobase we used a ramp protocol (from 0 to +250 pA, with a duration of 500 ms). Action potential (AP) firing frequency and number were obtained from the input/output (I/O) curve (from 0 to +400 pA, in 50 pA increments, with a duration of 500 ms). To assess the role of serotonin (5‐HT) and its receptors we perfused 5‐HT (30 μM, Cat. No. 3547), the antagonist of 5‐HT_3_ receptor Tropisetron (TROPI; 2 nM, Cat. No. 2459) or the antagonist of 5‐HT_1A_, (S)‐WAY 100135 (S‐WAY; 5 μM, Cat. No. 1253). In specific experiments, the antagonist of GABA_A_ receptors picrotoxin (PTX; 20 μM, Cat. No. 1128) was used. All the drugs were obtained from Tocris Bioscience.

### 2.5 Statistic Analysis

GraphPad Prism 8 was used for statistical analyses. Comparisons between control (−CRS) and chronic restraint stress (+CRS) groups were performed with a Student's *t*‐test. Two‐way ANOVA with repeated measures followed by Bonferroni's *post‐hoc* test was used for weekly running distance analysis. Two‐way ANOVA (stress × treatment) followed by *post‐hoc* Tukey's multiple comparison test was used for behavioural analysis. One‐way ANOVA with repeated measures followed by Tukey's multiple comparison test and Two‐way ANOVA with repeated measures (drug × current) followed by *post‐hoc* Tukey's multiple comparison tests were used for 5‐HT electrophysiological analysis. A significance level of 0.05 was set for all analyses. All values are shown as mean ± SEM.

## 3 Results

### 3.1 CRS Elicits Decreased Food Intake and Body Weight Gain

To determine the effect of exercise on the excitability of GCs after chronic stress, we first exposed the mice to either control (−CRS; *n* = 24) or chronic restraint stress (+CRS; *n* = 24) conditions for 14 days during 2 h/day (Figure 1a). Food intake was evaluated before (days 1–7) and during the exposition (days 8–21) to either control or CRS conditions. All animals had similar food intake (Figure 1b; *t*_(45)_ = 0.2919, *p* = 0.7717) and body weight (Figure 1e; *t*_(45)_ = 0.5609, *p* = 0.5776) before exposition to control or CRS conditions. However, CRS induced a significant reduction in total food intake from day 12 to 21 (Figure 1c; *t*_(45)_ = 4.848, *p* < 0.0001), which was reduced over time (Figure 1d; *F*_(1, 45)_ = 23.50, *p* < 0.0001; Two‐way ANOVA repeated measures). Additionally, CRS induced a significantly lower percentage of body weight gain from day 10 to 21 compared to −CRS mice (Figure 1f; *t*_(45)_ = 6.762, *p* < 0.0001), a difference that was increased over time (Figure 1g; *t*_(45)_ = 6.762, *p* < 0.0001). It is important to highlight that the difference in body weight gain between −CRS and +CRS mice was observed earlier than the reduction in food intake suggesting additional mechanisms to food intake involved in this process (Figure 1d,g). Altogether these findings suggest that exposure to chronic stress influences feeding behaviour and body weight regulation.

### 3.2 Running Mitigates the Altered Behaviour Responses Induced by CRS

**FIGURE 2:**
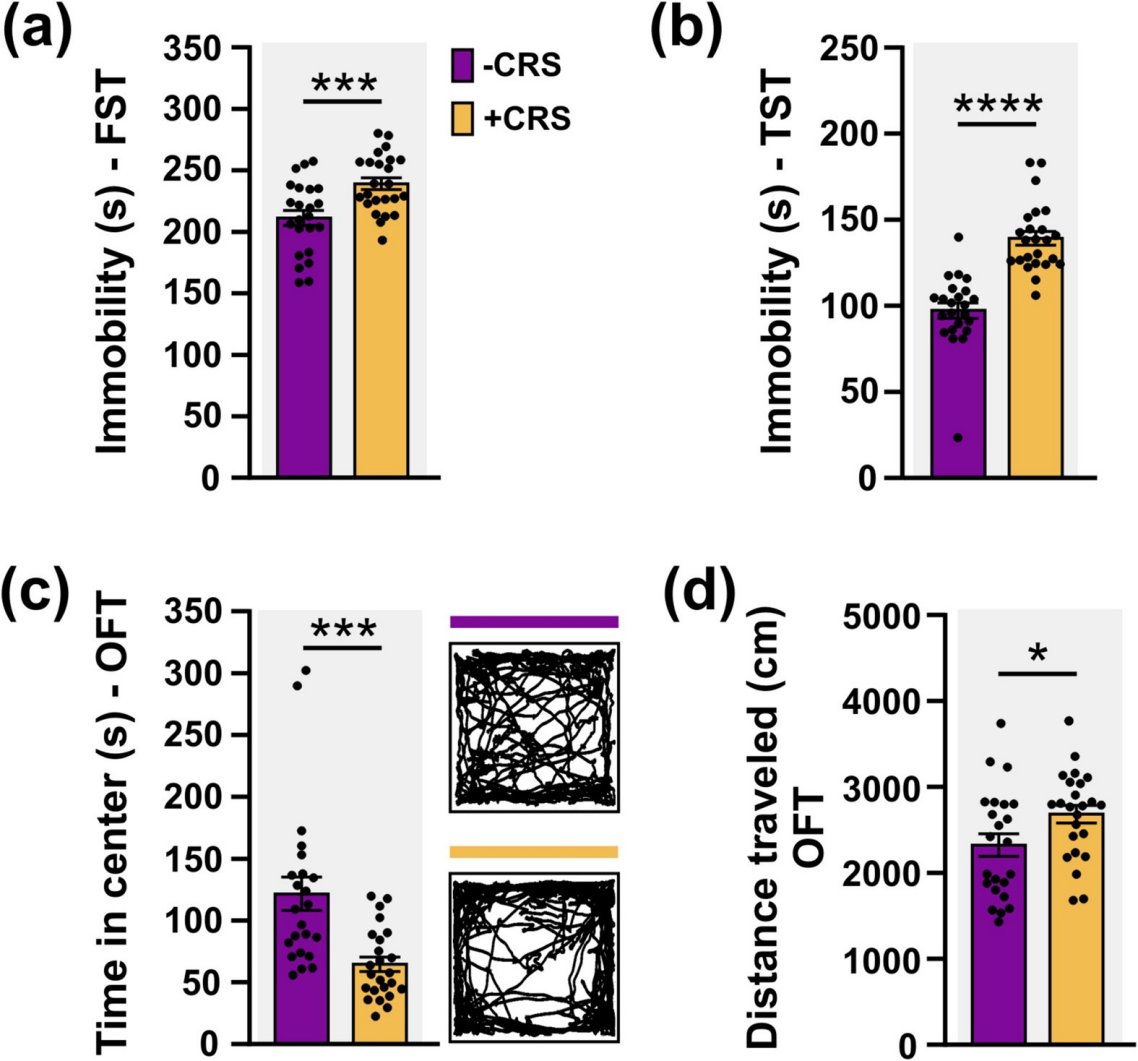
Chronic restraint stress promotes altered behavioural responses. (a), (b) Chronic restraint stress (+CRS) significantly increased immobility time in both the forced swimming test (FST) (a) and tail suspension test (TST) (b). (c) Mice exposed to +CRS spend less time in the centre of the open field arena compared to non‐stressed (−CRS) mice. Right: Example of path traces during the open field test (OFT) of mice exposed to either −CRS (purple line) or +CRS (yellow line) conditions. (d) Distance travelled in the OFT was significantly increased in mice exposed to +CRS. +CRS, *n* = 24; −CRS, *n* = 23. Unpaired *t*‐test, 2‐tailed was used for comparison. Values are mean ± SEM. Statistical significances are denoted by *p ≤ 0.05, ***p ≤ 0.001, **** *p* ≤ 0.0001.

Next, we evaluated the effects of CRS on behaviour (PRE, Figure 1a). We evaluated coping strategies during acute inescapable stress using the forced swimming test (FST) and tail suspension test (TST). CRS significantly increased immobility time in the FST (*t*_(45)_ = 3.586, *p* = 0.0008) and TST (*t*_(45)_ = 6.995, *p* < 0.0001) (Figure 2a,b). Avoidance behaviours were assessed using the open field test (OFT). CRS significantly reduced the time in the centre of the arena (*t*_(45)_ = 3.951, *p* = 0.0003) and increased the distance travelled (*t*_(45)_ = 2.137, *p* = 0.0380) (Figure 2c,d). Overall, this shows that CRS elicits altered behavioural responses.

**FIGURE 3:**
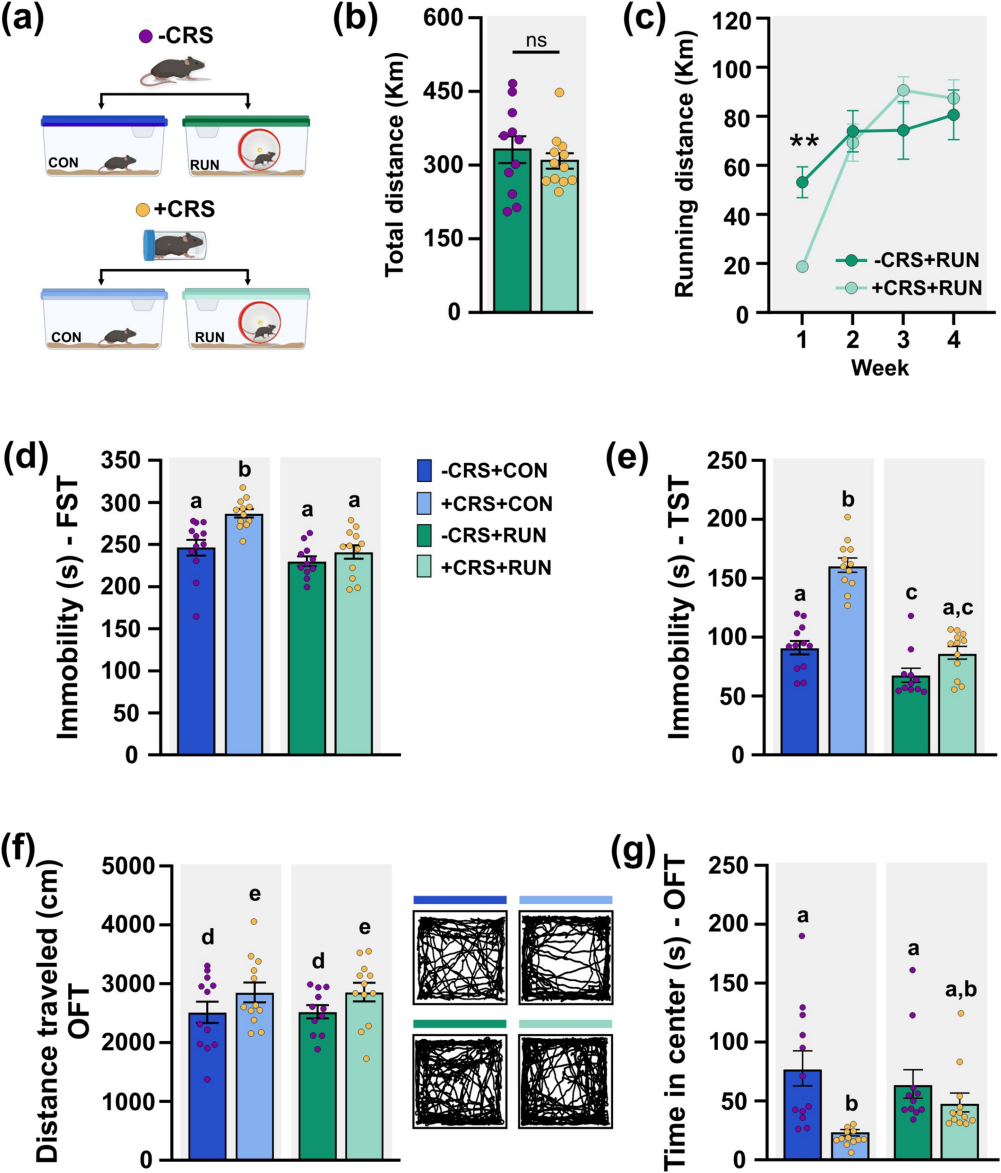
Running mitigates the altered behavioural responses elicited by CRS. (a) Adult young mice were exposed to chronic restraint stress (+CRS) or control non‐stressed conditions (−CRS) for 14 days. Thereafter, mice were housed under either control (CON) or running (RUN) conditions for 30 days. (b) The total distance run by +CRS and −CRS mice was similar. (c) Weekly distance run by mice from +CRS and −CRS groups. During the first week, mice exposed to CRS ran significantly less than mice from the −CRS group. (d), (e) Running decreases the immobility time in mice exposed to CRS in the forced swimming test (FST) (d) and tail suspension test (TST) (e). (f) Open field test (OFT) shows that CRS increases the distance travelled. −CRS + CON, *n* = 12; +CRS + CON, *n* = 12; −CRS + RUN, *n* = 11; +CRS + RUN, *n* = 12. Right: Representative open field path traces of mice exposed to −CRS + CON (dark blue), +CRS + CON (light blue), −CRS + RUN (dark green), or +CRS + RUN (light green) conditions. (g) Chronic restraint stress significantly reduces the time in the centre of the arena, while chronically stressed mice exposed to running spend similar time in the centre of the arena than the non‐stressed control (−CRS + CON) and runner (−CRS + RUN) mice. Values are mean ± SEM. For comparisons, it was used in *(b)* an unpaired *t*‐test, 2‐tailed, in (c) a two‐way ANOVA with repeated measures followed by Bonferroni's *post‐hoc* test and *(d) ‐ (g)* two‐way ANOVA followed by Tukey's *post‐hoc* test. Different letters in columns indicate significant differences as follows: ^a,b,c^ *p* < 0.05 *stress* × *treatment* interaction; ^d,e^ *p* < 0.05 main effect of *stress*.

Mice from −CRS and +CRS groups were randomly subdivided into two groups and housed under control (CON: −CRS + CON, + CRS + CON) or running (RUN: −CRS + RUN, + CRS + RUN) conditions for 30 days (Figures 1a, and 3a). Non‐stressed (−CRS) and chronically stressed (+CRS) mice ran similar distances over the 30 days (*t*_(21)_ = 0.7506, *p* = 0.4612) (Figure 3b). However, a per‐week analysis showed that during the first week, +CRS mice ran significantly shorter distance than −CRS mice (Two‐way ANOVA repeated measures, stressor × treatment *F*_(3, 63)_ = 5.996, *p* = 0.0012, *post‐hoc* Bonferroni's test, Week 1 *p* = 0.0092) (Figure 3c).

The effect of running on CRS‐induced behaviours was evaluated with the same battery of tests (POST, Figure 1a). Two‐way ANOVA analysis showed that chronically stressed mice, exposed to 30 days of sedentary control conditions (+CRS + CON), exhibited increased immobility time in the FST and TST compared to non‐stressed mice (−CRS + CON) showing that CRS induces long‐lasting behavioural alterations (Figure 3d,e; FST: stressor × treatment *F*_(1, 43)_ = 3.740, *p* = 0.0489, *post‐hoc* Tukey's test −CRS + CON vs. +CRS + CON, *p* = 0.0016; TST: stressor × treatment *F*_(1, 43)_ = 19.55, *p* < 0.0001; *post‐hoc* Tukey's test −CRS + CON vs. +CRS + CON, *p* < 0.0001). However, in chronically stressed mice exposed to 30 days of running (+CRS + RUN), the immobility time was significantly reduced compared to untreated mice (+CRS + CON) (Figure 3d,e; FST: +CRS + CON vs. +CRS + RUN, *p* = 0.0004; TST: +CRS + CON vs. +CRS + RUN, *p* < 0.0001). No significant difference was found between chronically stressed runner mice and non‐stressed runner mice (Figure 3d,e; FST: −CRS + RUN vs. +CRS + RUN, *p* = 0.7361; TST: −CRS + RUN vs. +CRS + RUN, *p* = 0.1188) suggesting that running mitigates the behavioural alterations induced by CRS.

In addition, OFT showed that mice exposed to chronic stress followed by 30 days of control sedentary or running conditions showed a significantly higher distance travelled compared to non‐stressed mice (Figure 3f; Two‐way ANOVA, main effect of stressor *F*_(1, 43)_ = 4.421, *p* = 0.0414; stressor × treatment *F*_(1, 43)_ = 0.0005, *p* = 0.9812). However, stressed mice housed in control sedentary conditions spent significantly less time in the centre of the arena compared to non‐stressed mice (Figure 3g; Two‐way ANOVA, stressor × treatment *F*_(1, 43)_ = 4.269, *p* = 0.0449; *post‐hoc* Tukey's test −CRS + CON vs. +CRS + CON *p* = 0.0012). While +CRS running mice spent similar time in the centre of the arena than non‐stressed mice (Figure 3g; −CRS + RUN vs. +CRS + RUN, *p* = 0.7142; −CRS + CON vs. +CRS + RUN, *p* = 0.2045) suggesting that running mitigates the altered avoidance behaviour induced by CRS. Altogether suggests that running mitigates the altered behaviours triggered by CRS.

### 3.3 One Month After CRS, the Basal Passive and Active Membrane Properties of vDG GCs Remain Unchanged

**FIGURE 4:**
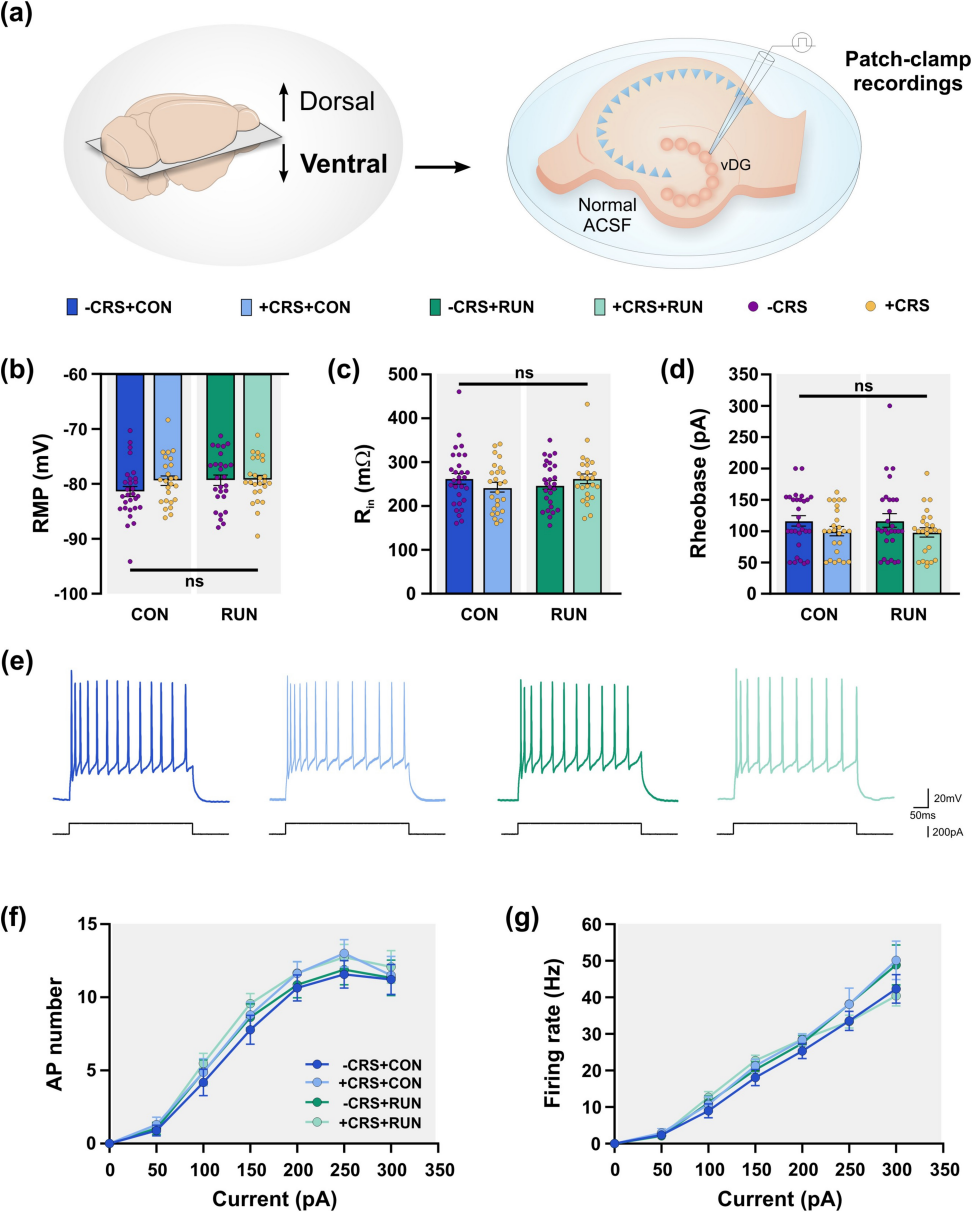
One month after chronic restraint stress (CRS) basal passive and active membrane properties of granule cells are unchanged. (a) Whole‐cell patch‐clamp recordings were performed in granule cells (GCs) located in the ventral dentate gyrus (vDG) under basal conditions (ACSF). (b) The basal resting membrane potential (RMP) is not modified in GCs by CRS and running. (c) Chronic restraint stress and running do not modify the basal input resistance (R_in_) of GCs located in the vDG. (d) The basal rheobase of GCs is not modified by CRS and running. (e) Representative traces of the membrane potential in response to 200 pA of current injection from GCs of mice from −CRS + CON (dark blue), +CRS + CON (light blue), −CRS + RUN (dark green) and +CRS + RUN (light green) groups. (f) Mean action potentials (AP) number from GCs as a function of current injection of increasing magnitude. No significant differences were observed between groups. (g) Graph of the mean firing rate of AP in response to current injections of increasing magnitude in granule cells. There was no significant difference in the firing rate between groups. Cells number × group: CRS + CON, *n* = 30 from 12 mice; +CRS + CON, *n* = 26 from 10 mice; −CRS + RUN, *n* = 33 from 12 mice; +CRS + RUN, *n* = 28 from 12 mice. Values are mean ± SEM. For (b), (c) and (d), two‐way ANOVA was used for comparison. No significant main effect or interaction. For (f) and (g), two‐way ANOVA with repeated measures was used for comparison. No significant main effect of group or interaction. Not significant denoted by ns, *p* > 0.05. See Table S1 for details of statistical values.

The GC excitability may be a key factor for stress resilience (Anacker et al. 2018). To begin to elucidate the mechanisms underlying the running‐inducing mitigating effects in the altered behaviours triggered by CRS, we performed *in vitro* whole‐cell patch‐clamp recordings of vDG GCs under basal conditions (ACSF). Brain slices were derived from mice in either non‐stressed (−CRS) conditions or exposed to CRS for 14 days (+CRS), and subsequently housed for 30 days under either CON or RUN conditions (Figure 4a). We evaluated the passive and active membrane properties of the vDG GCs. Statistical analysis showed that the properties of the GCs from chronically stressed mice exposed to control sedentary conditions were unchanged compared to non‐stressed control mice (Two‐way ANOVA, stressor × treatment; Table S1 for statistical values for the main effect of stress, and the main effect of treatment). Specifically, basal resting membrane potential (RMP; *F*_(1,102)_ = 1.162, *p* = 0.2837; Figure 4b), input resistance (R_in_; *F*_(1,102)_ = 1.178, *p* = 0.2804; Figure 4c) and rheobase (*F*_(1,102)_ = 0.0000023, *p* = 0.9988; Figure 4d), as well as basal GC excitability, measured by the maximum number of (APs; repeated measures group × current *F*_(18,588)_ = 0.2487; *p* = 0.9994; Figure 4e,f), and firing rate, were unchanged (repeated measures group × current *F*_(18,588)_ = 1.112; *p* = 0.3354) (Figure 4g). Moreover, running did not modify the basal passive and active membrane properties of the GCs from non‐stressed and chronically stressed mice (Figure 4b–g). Taken together, these results show that the basal membrane properties of the vDG GCs are not modified by CRS or running.

### 3.4 Running Recovers 5‐HT Modulation in GCs From Mice Exposed to CRS

**FIGURE 5:**
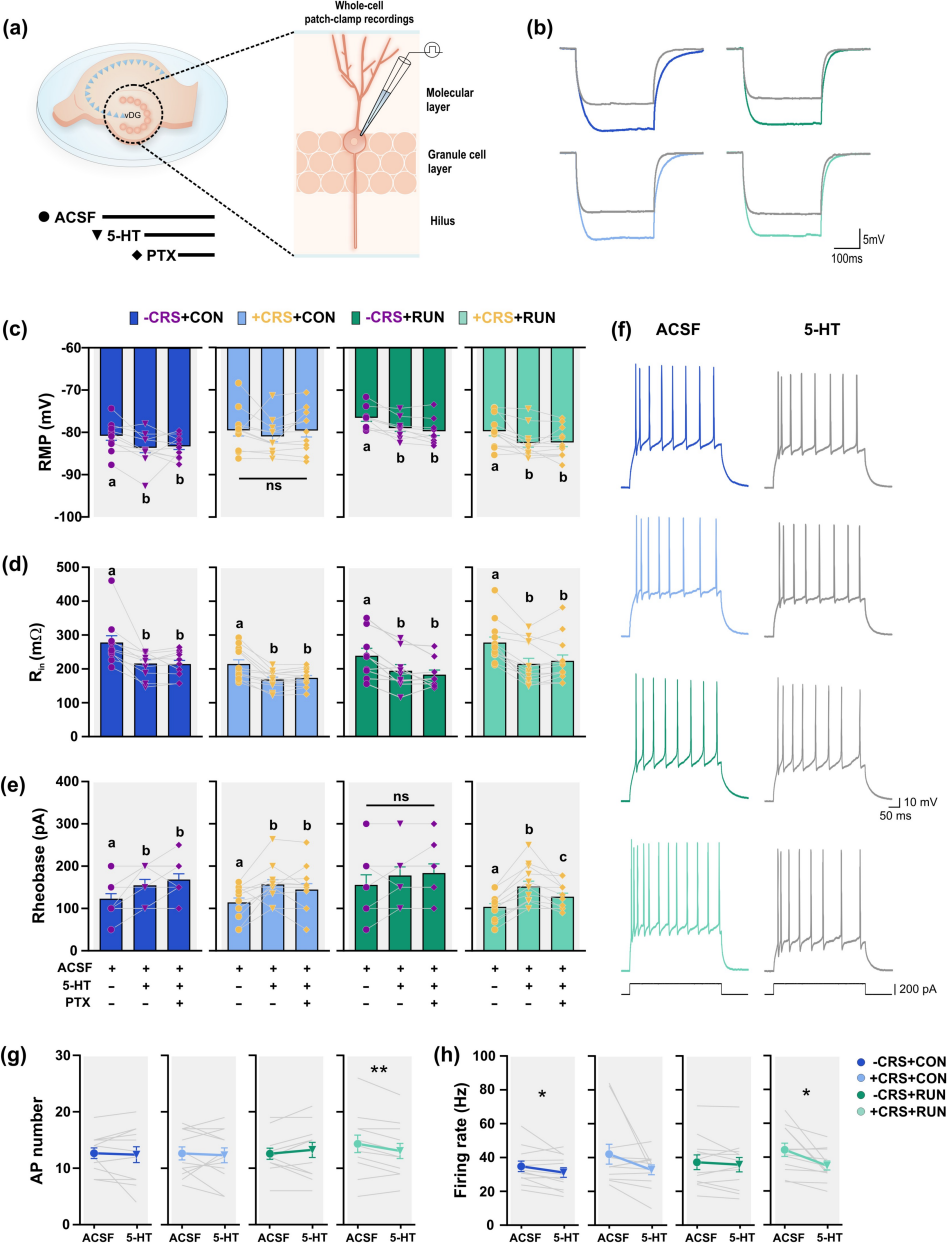
Running recovers CRS‐induced loss of serotonergic modulation of GC excitability. (a) Whole‐cell patch‐clamp recordings were performed in granule cells (GCs) located in the outer granule cell layer of the ventral dentate gyrus (vDG) in the sequential bath application of artificial cerebrospinal fluid (ACSF), 5‐HT (30 μM) and picrotoxin (20 μM). (b) Representative traces of membrane potential of GCs in response to a hyperpolarized pulse (100 pA) under ACSF (colour) and 5‐HT (grey) bath perfusion from −CRS + CON (dark blue), +CRS + CON (light blue), CRS + RUN (dark green) and +CRS + RUN (light green) mice. (c) Bath application of 5‐HT reduces significantly the resting membrane potential (RMP) of GCs from unstressed control (−CRS + CON), unstressed runner (−CRS + RUN) and stressed runner (+CRS + RUN) mice, but not from stressed untreated (+CRS + CON) mice. The blocking of GABA_A_ receptors with PTX does not modify the RMP in any of the groups. (d) Summary bar graphs show that 5‐HT significantly reduces the input resistance (R_in_) of GCs in all the groups. PTX does not block the 5‐HT modulation of R_in_ in any of the groups. (e) Bar graph shows that 5‐HT significantly increases the rheobase of GCs from −CRS + CON, +CRS + CON and +CRS + RUN mice but not from −CRS + RUN mice. Blocking of GABA_A_ receptors with PTX blocked partially the 5‐HT modulation of the rheobase in CGs from +CRS + RUN mice. PTX does not block the 5‐HT modulation of the rheobase in GCs from −CRS + CON, +CRS + CON and −CRS + RUN mice. (f) Representative traces of membrane potential of GCs in response to current injection (200 pA) under ACSF (group colour) and 5‐HT (grey) conditions from −CRS + CON (dark blue), +CRS + CON (light blue), −CRS + RUN (dark green) and +CRS + RUN (light green) mice. (g) Summary graph showing that 5‐HT only reduces the number of action potentials (AP) in GCs from +CRS + RUN mice. (h) Plots show that 5‐HT significantly reduces the firing rate of GCs from −CRS + CON and +CRS + RUN mice. Cell number × group in (c), (d) and (e): CRS + CON, *n* = 11 from 8 mice; +CRS + CON, *n* = 13 from 5 mice; −CRS + RUN, *n* = 9 from 6 mice; +CRS + RUN, *n* = 13 from 8 mice. Cell number × group in (g) and (h): CRS + CON, *n* = 12 from 10 mice; +CRS + CON, *n* = 12 from 5 mice; −CRS + RUN, *n* = 12 from 10 mice; +CRS + RUN, *n* = 12 from 7 mice. Values are mean ± SEM. For (c), (d) and (e)*,* one‐way ANOVA with repeated measures followed by Tukey's *post‐hoc* test was used for comparisons. For (g) and (h), paired *t*‐tests 2‐tailed were used for comparisons. Different letters (a, b, c) in columns indicate significant differences (*p* < 0.05). See Table S2 for details of statistical values.

Next, we assessed the influence of serotonergic modulation on the passive and active membrane properties of the vDG GCs (Figure 5a). One‐way ANOVA analysis followed by the *post‐hoc* Tukey's multiple comparisons tests showed that in GCs from non‐stressed control mice bath application of 30 μM of 5‐HT produced a significant hyperpolarization of the RMP (*F*_(2, 20)_ = 6.321, *p* = 0.0075; ACSF vs. 5‐HT, *p* = 0.0106; Figure 5c), the reduction of the R_in_ (*F*_(2, 20)_ = 13.63, *p* = 0.0002; ACSF vs. 5‐HT, *p* = 0.0007; Figure 5b,d) and the increment of the rheobase (*F*_(2, 20)_ = 12.34, *p* = 0.0003; ACSF vs. 5‐HT, *p* = 0.0078, Figure 5e) resulting in decreased firing rate (*t*_(11)_ = 2.293, *p* = 0.0425; Figure 5h), without modifying the number of AP (*t*_(11)_ = 0.3093, *p* = 0.7629; Figure 5f,g). This indicates that 5‐HT reduces the excitability of the GCs under control conditions as has been observed in previous studies (Piguet and Galvan 1994). In GCs from chronically stressed sedentary control mice, 5‐HT significantly reduced the R_in_ (*F*_(2, 24)_ = 22.28, *p* < 0.0001; ACSF vs. 5‐HT, *p* < 0.0001; Figure 5b,d) and increased the rheobase (*F*_(2, 24)_ = 7.191, *p* = 0.0036; ACSF vs. 5‐HT, *p* = 0.0033; Figure 5e), however, the RMP (*F*_(2, 24)_ = 0.1436, *p* = 0.8670; ACSF vs. 5‐HT, *p* = 0.8994; Figure 5c), AP number (*t*_(11)_ = 0.3817, *p* = 0.7100; Figure 5f,g) and firing rate (*t*_(11)_ = 1.668, *p* = 0.1235; Figure 5h) were not modulated by 5‐HT suggesting that CRS precludes the serotonergic modulatory effect on the excitability of the GCs. This lack of serotonergic modulation on the excitability of GCs was also observed in non‐stressed runner mice; however, the properties were differentially modulated. Specifically, 5‐HT significantly hyperpolarized the RMP (*F*_(2, 16)_ = 16.02, *p* = 0.0002; ACSF vs. 5‐HT, *p* = 0.0020; Figure 5c) and decreased the R_in_ (*F*_(2, 16)_ = 13.03, *p* = 0.0004; ACSF vs. 5‐HT, *p* = 0.0039; Figure 5b,d) without modify the rheobase (*F*_(2, 16)_ = 3.294, *p* = 0.0634; ACSF vs. 5‐HT, *p* = 0.1597; Figure 5e), AP number (*t*_(11)_ = 1.017, *p* = 0.3308; Figure 5f,g) and firing rate (*t*_(11)_ = 0.9365, *p* = 0.3691; Figure 5h) suggesting a differential mechanism underlying 5‐HT modulation of GCs between CRS and running. Interestingly, running recovered the loss of 5‐HT modulation in GCs from mice exposed to CRS. Serotonin significantly hyperpolarized the RMP (*F*_(2, 24)_ = 11.08, *p* = 0.0004; ACSF vs. 5‐HT, *p* = 0.0010; Figure 5c), reduced the R_in_ (*F*_(2, 24)_ = 33.72, *p* < 0.0001; ACSF vs. 5‐HT, *p* < 0.0001; Figure 5b,d) and increased the rheobase (*F*_(2, 24)_ = 20.40, *p* < 0.0001; ACSF vs. 5‐HT, *p* < 0.0001; Figure 5e) resulting in a significant reduction of AP number (*t*_(11)_ = 3.362, *p* = 0.0063; Figure 5f,g) and firing rate (*t*_(11)_ = 2.780, *p* = 0.0179; Figure 5h).

GABAergic interneurons are the major target of the median raphe, and their serotonergic activation may influence the excitability of GCs (Halasy et al. 1992; Kawa 1994). Therefore, we evaluated whether the application of the GABA_A_ receptor antagonist, picrotoxin (PTX; 20 μM) at a concentration that blocks the phasic activation of GABA_A_ receptors, blocks the serotonergic modulation of GCs membrane properties. The 5‐HT modulation of the RMP, R_in_ and rheobase was not abolished by the application of PTX in non‐stressed control (−CRS + CON), stressed control (+CRS + CON) and non‐stressed runner mice (−CRS + RUN) (Figure 5c–f, Table S2). However, in stressed runner mice (+CRS + RUN), the serotonergic modulation of the rheobase was significantly blocked by PTX (5‐HT vs. 5‐HT + PTX, *p* = 0.0108), although the serotonergic modulation was partially blocked as the rheobase was significantly different from the one observed in ACSF conditions (ACSF vs. 5‐HT + PTX, *p* = 0.0102) (Figure 5e). Altogether our findings suggest that running recovers the serotonergic modulation of GCs passive and active membrane properties which may be partially mediated by the activation of GABAergic interneurons.

### 3.5 Running Recovers the Activation of 5‐HT_1A_ Receptors in GCs and Promotes the Activation of 5‐HT_3_ Receptors in the Dentate Gyrus

**FIGURE 6:**
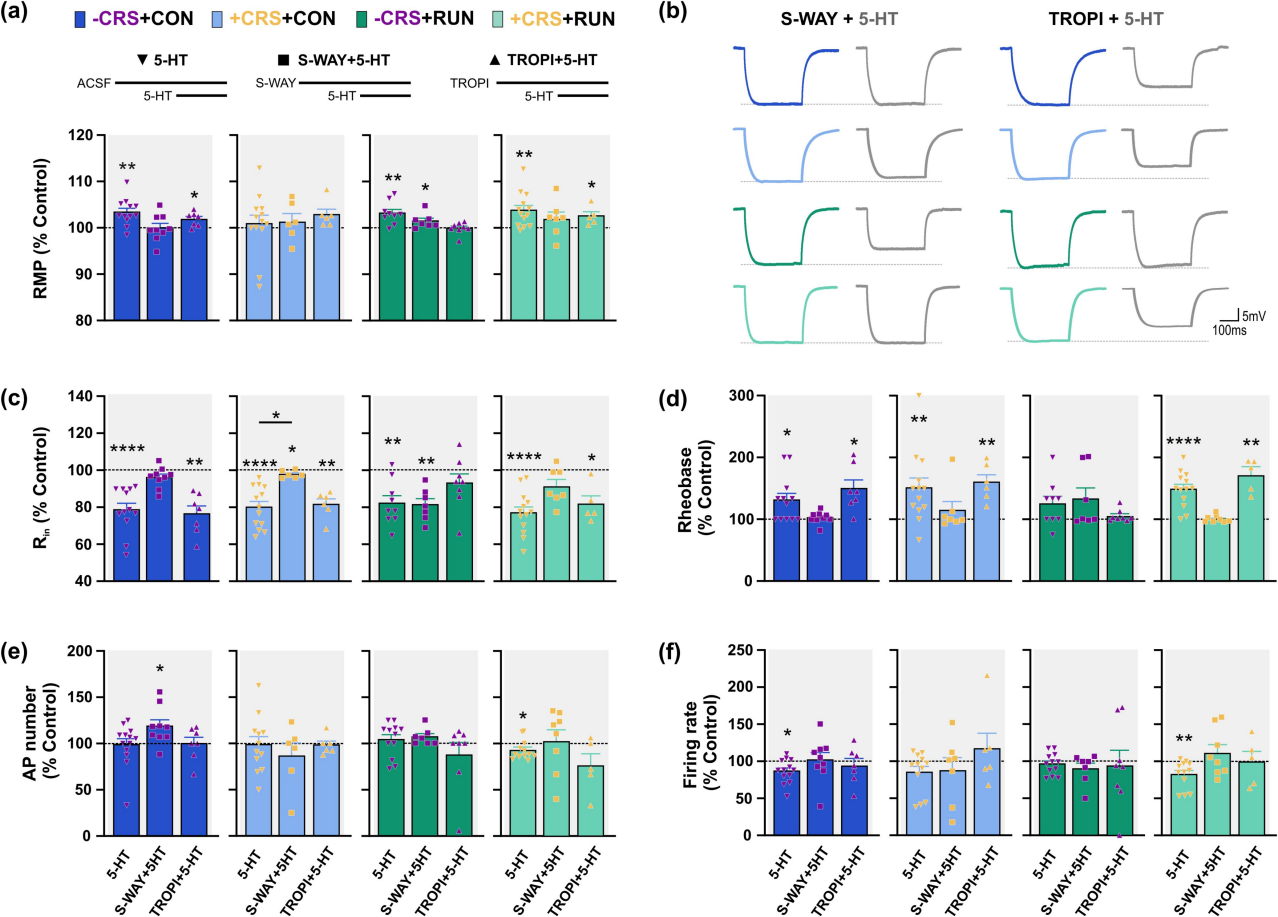
CRS‐induced loss of 5‐HT_1A_ receptor activation is recovered by running. (a), (c–f) Summary bar graphs showing the percentage of changes of RMP (a), R_in_ (c), rheobase (d), AP number (e) and firing rate (f) of GCs in response to the bath application of 5‐HT (30 μM), the antagonists of 5‐HT_1A_ receptor, S‐WAY (5 μM) and 5‐HT_3_ receptor, TROPI (2 nM) in independent experiments. The responses correspond to their specific control: 5‐HT: ACSF vs. 5‐HT; S‐WAY+5‐HT: S‐WAY vs. S‐WAY + 5‐HT; TROPI + 5‐HT: TROPI vs. TROPI + 5‐HT. (b) Representative traces depicting the GC membrane potential in response to a hyperpolarized pulse (100 pA) in the presence of S‐WAY (group colour) + 5‐HT (grey) or TROPI (group colour) + 5‐HT (grey) from −CRS + CON (dark blue), +CRS + CON (light blue), −CRS + RUN (dark green) and +CRS + RUN (light green) mice. Values are mean ± SEM. For comparison, it was used paired *t*‐test, 2‐tailed. Statistical significances are denoted by *p ≤ 0.05, **p ≤ 0.01, ****p ≤ 0.0001. See Table S3 for details of statistical values.

**FIGURE 7:**
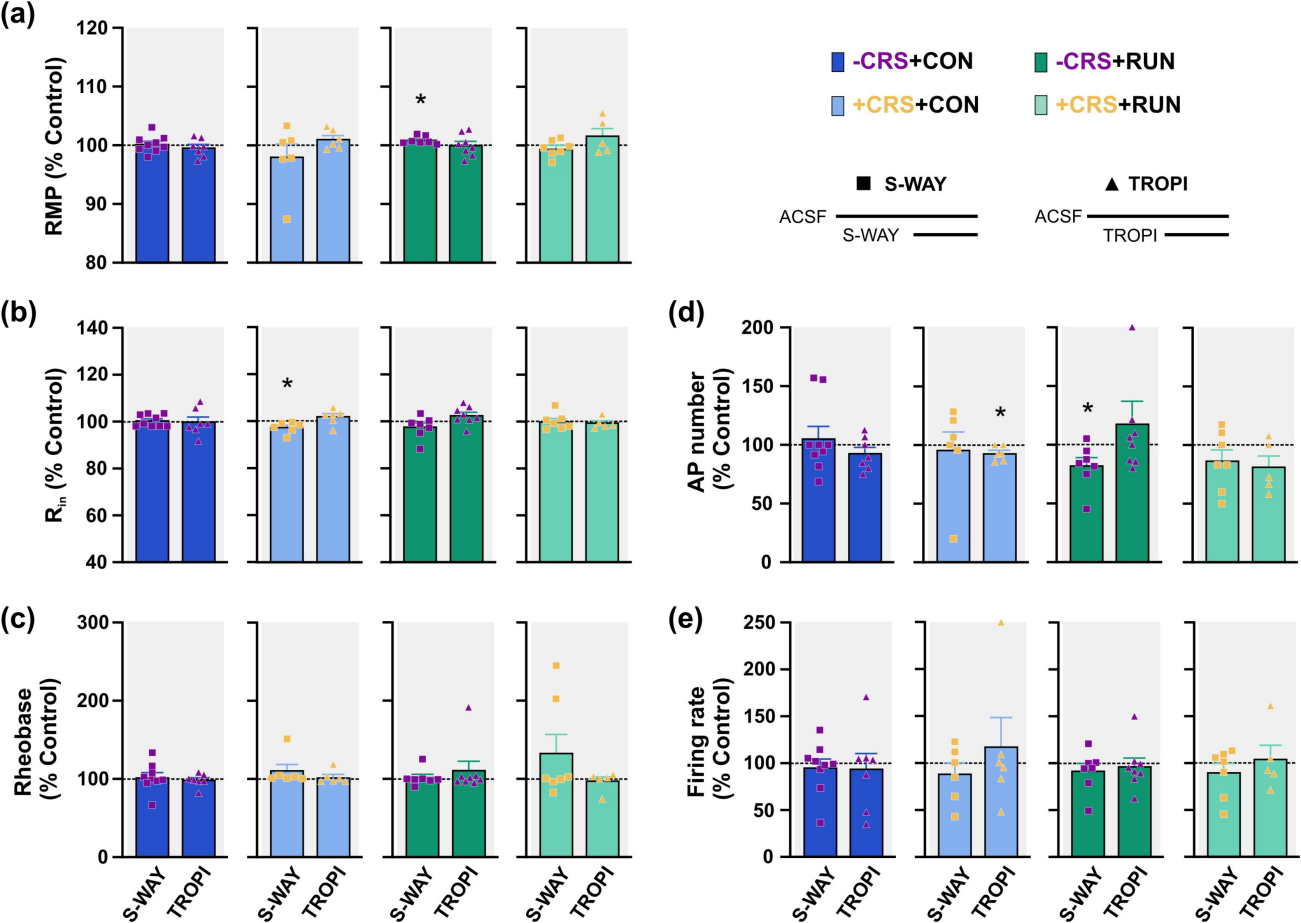
Chronic restraint stress and running induce the tonic activation of 5‐HT_1A_ and 5‐HT_3_ receptors. (a)–(e) Summary bar graphs showing the percentage of changes of RMP (a), R_in_ (b), rheobase (c), AP number (d) and firing rate (e) of GCs in response to the bath application of S‐WAY only (5 μM) and TROPI only (2 nM) compared to ACSF in independent experiments. S‐WAY: ACSF vs. S‐WAY; TROPI: ACSF vs. TROPI. See Table S4 for details of statistical values. Values are mean ± SEM. For comparison, paired *t*‐tests of 2‐tailed were used. Statistical significances are denoted by *p ≤ 0.05.

The 5‐HT_1A_ and 5‐HT_3_ receptors have been implicated in chronic stress and exercise‐induced effects (Gomez‐Merino et al. 2001; Kondo et al. 2015; Samuels et al. 2015; Pietrelli et al. 2018; Bickle et al. 2024). Therefore, to further identify the mechanisms underlying the anxiolytic and stress coping effect of running, we evaluated 5‐HT modulation of the passive and active membrane properties in the presence of 5‐HT_1A_ and 5‐HT_3_ receptors antagonists, (S)‐WAY 100135 (S‐WAY) and Tropisetron (TROPI) respectively, in independent experiments. Specifically in GCs from non‐stressed control mice (−CRS + CON), paired *t*‐test analysis showed that S‐WAY (S‐WAY vs. SWAY+5‐HT) significantly prevents the serotonergic modulation of RMP (*t*_(8)_ = 0.01679, *p* = 0.9870), R_in_ (*t*_(8)_ = 2.150, *p* = 0.0638) and rheobase (*t*_(8)_ = 0.7598, *p* = 0.4692) (Figure 6a–d), whereas TROPI (TROPI vs. TROPI+5‐HT) did not prevent the 5‐HT modulation of RMP (*t*_(6)_ = 3.283, *p* = 0.0168), R_in_ (*t*_(6)_ = 5.675, *p* = 0.0013) and rheobase (*t*_(6)_ = 3.552, *p* = 0.0120) (Figure 6a–d; Table S3). Moreover, S‐WAY+5‐HT significantly increased the AP number (*t*_(8)_ = 2.718, *p* = 0.0263) and prevented the reduction of the firing rate induced by 5‐HT (*t*_(8)_ = 0.1823, *p* = 0.8599) (Figure 6e,f; Table S3) suggesting that in control conditions 5‐HT modulates the excitability of the GCs through the direct activation of 5‐HT_1A_ receptors. Interestingly, TROPI also prevented the reduction of the firing rate induced by 5‐HT (TROPI vs. TROPI+5‐HT; *t*_(6)_ = 0.5971, *p* = 0.5723; Figure 6f) suggesting an indirect modulation since the direct activation of 5‐HT_3_ receptors would increase neuronal excitability instead of reducing it (Barnes et al. 2009). When only S‐WAY or TROPI were perfused, before the exogenous application of 5‐HT, no modifications were observed in the GCs membrane properties of −CRS + CON mice (Figure 7a–e; Table S4). Altogether, our data suggests that under control conditions 5‐HT modulates the GC excitability through the direct activation of 5‐HT_1A_ receptors and the indirect modulation of the excitability through 5‐HT_3_ receptor activation.

In GCs from chronically stressed untreated mice (+CRS + CON), S‐WAY prevented the 5‐HT modulation of rheobase (*t*_(5)_ = 1.064, *p* = 0.3362) whereas R_in_ modulation was not prevented (*t*_(5)_ = 2.679, *p* = 0.0439; Figure 6c). However, when 5‐HT vs. S‐WAY+5‐HT modulatory effects in R_in_ were compared, it showed significant differences (*t*_(17)_ = 3.787; *p* = 0.0015, unpaired *t*‐test) suggesting that S‐WAY prevented partially the 5‐HT modulation. No changes were observed in RMP, AP number and firing rate since CRS does not induce serotonergic modulation in these properties (Figures 5c,g,f and 6a,e,f; Table S3). Furthermore, when only S‐WAY was applied, R_in_ was slightly but significantly reduced (*t*_(5)_ = 2.693, *p* = 0.0431; Figure 7b) suggesting that CRS induces the tonic 5‐HT_1A_ receptor activation. Moreover, the reduction in R_in_ also suggests an indirect modulation, as the blocking of 5‐HT_1A_ receptors in GCs would induce an increment of R_in_ (Piguet and Galvan 1994). On the other hand, TROPI did not prevent the 5‐HT modulation of R_in_ (*t*_(5)_ = 6.235, *p* = 0.0016) and rheobase (*t*_(5)_ = 4.876, *p* = 0.0046) (Figure 6c,d). However, when only TROPI was bath applied, the AP number was significantly reduced (*t*_(5)_ = 2.767, *p* = 0.0395; Figure 7d) suggesting an indirect modulation of GCs excitability through the tonic activation of 5‐HT_3_ receptors. No modifications were observed in RMP, R_in_, rheobase and firing rate when only TROPI was applied (Figure 7a–c,e; Table S4). Altogether our findings indicate that CRS alters the serotonergic modulation of GC excitability reducing the activation of 5‐HT_1A_ receptors in GC and inducing the indirect modulation of GCs through the tonic activation of 5‐HT_1A_ and 5‐HT_3_ receptors.

In GCs from non‐stressed runner mice (−CRS + RUN), S‐WAY did not prevent the 5‐HT modulation of RMP (*t*_(6)_ = 2.460, *p* = 0.0491) and R_in_ (*t*_(6)_ = 5.649, *p* = 0.0013) (Figure 6a–c). However, when only S‐WAY was bath applied, the RMP was significantly hyperpolarized (*t*_(6)_ = 3.398, *p* = 0.0145) and the AP number was significantly reduced (*t*_(6)_ = 2.532, *p* = 0.0446) (Figure 7a,d) suggesting the indirect modulation of the GCs excitability through the tonic activation of 5‐HT_1A_ receptors. TROPI prevented the hyperpolarization of the RMP (*t*_(7)_ = 0.0891, *p* = 0.9315) and the reduction of the R_in_ (*t*_(7)_ = 1.410, *p* = 0.2014) induced by 5‐HT (Figure 6a–c) suggesting an indirect modulation of GCs since the direct blocking of 5‐HT_3_ receptors would induce the opposite effects in GCs. The rheobase, AP number and firing rate were not modulated by 5‐HT and no changes were observed when S‐WAY + 5‐HT and TROPI + 5‐HT were applied (Figures 5e,g,h and 6d–f; Table S3). Thus, running may modulate the excitability of GCs through two indirect serotonergic modulatory mechanisms: the activation of 5‐HT_3_ receptors, as has been suggested (Kondo et al. 2015), and the tonic activation of 5‐HT_1A_ receptors.

When CRS was followed by running treatment, the serotonergic modulation through 5‐HT_1A_ receptors was recovered since S‐WAY (S‐WAY vs. S‐WAY + 5‐HT) prevented the modulation of RMP (*t*_(6)_ = 1.395, *p* = 0.2126), R_in_, (*t*_(6)_ = 2.375, *p* = 0.0551) and rheobase (*t*_(6)_ = 1.295, *p* = 0.2520), while TROPI was unable to block 5‐HT modulation of these properties (Figure 6a–d; Table S3). Running also recovered the serotonergic modulation of GC's firing rate that was lost by CRS. This effect was mediated by the activation of 5‐HT_1A_ and 5‐HT_3_ receptors, as S‐WAY (*t*_(7)_ = 0.9737, *p* = 0.3626) and TROPI (*t*_(4)_ = 0.0771, *p* = 0.9422) prevented the 5‐HT‐induced reduction in the firing rate (Figure 6f). Interestingly, in stressed mice, running induces the 5‐HT modulation of AP number, which is not observed in unstressed control or runner mice (Figure 5g). This 5‐HT modulatory effect was also prevented by S‐WAY (*t*_(7)_ = 0.2178, *p* = 0.8338) and TROPI (*t*_(4)_ = 1.854, *p* = 0.1374) (Figure 6e). The modulation through the 5‐HT_3_ receptor, however, may be indirectly mediated since the direct 5‐HT_3_ receptor block would induce an opposite effect on the excitability of GCs. No effect was observed when only S‐WAY or TROPI were baths applied (Figure 7a–e; Table S4). Thus, running may mitigate the altered behavioural responses to CRS through two serotonergic modulatory mechanisms: rescuing the activation of 5‐HT_1A_ receptors in GCs and activating 5‐HT_3_ receptors that indirectly modulate GCs excitability.

## 4 Discussion

Physical exercise ameliorates stress‐related behaviours such as depression and anxiety (Singh et al. 2023). However, the underlying mechanisms are largely unknown. Our results show that 30 days of running mitigates the behavioural alterations promoted by CRS. Electrophysiological recordings revealed that CRS modifies the serotonergic modulation of GC's excitability. Specifically, CRS reduced the activation of 5‐HT_1A_ receptors in GCs and induced the tonic activation of 5‐HT_1A_ and 5‐HT_3_ receptors that indirectly modulate GC's excitability by 5‐HT. Thirty days of running after CRS recovered the activation of 5‐HT_1A_ receptors in GCs and induced the indirect modulation of GC's excitability through the 5‐HT_3_ receptors activation. Thus, 5‐HT_1A_ and 5‐HT_3_ receptors may play an important role in exercise‐induced regulation of chronic stress.

Chronic stress induces a series of alterations at physiological, behavioural, cellular and molecular levels that lead to mental disorders (McEwen et al. 2015; McEwen, Nasca and Gray 2016; Tran and Gellner 2023). CRS in mice causes altered stress coping, and avoidance behaviours, as well as physiological modifications (Kim and Han 2006; Jeong, Lee and Kang 2013). Here we show that CRS rapidly induces a reduction in food intake and body weight gain as has been observed previously (Kim and Han 2006; Jeong, Lee and Kang 2013). Studies have shown these changes parallel with the increment of corticosterone levels (Kim and Han 2006; Jeong, Lee and Kang 2013). Reduced body weight gain was observed even earlier than diminished food intake suggesting additional mechanisms. Indeed, it has been shown that CRS induces, from day 1, changes in the expression of metabolism‐related genes and hypothalamic genes related to body weight control which may lead to a reduction in body weight gain (Ricart‐Jané et al. 2005; Sato et al. 2006; Jeong, Lee and Kang 2013). Furthermore, CRS induces atrophic gene expression and loss of muscle mass (Allen et al. 2010) which may explain the lower running performance during the first week in chronically stressed mice. However, their performance recovered during the second week to similar levels as the non‐stressed mice. Our results show that 14 days of CRS induces long‐lasting altered behavioural responses. Increased passive coping behaviour (immobility time) in FST and TST as well as increased avoidance behaviour (reduction of time in the centre of the arena) in OFT were observed 1 month after CRS exposure in sedentary mice. Running ameliorated the altered behaviours observed after CRS. Our results are consistent with previous studies in rodents showing that exercise prevents and/or ameliorates the altered behavioural responses induced by CRS (Kim and Leem 2014; Kang et al. 2020; Xiao et al. 2021).

We observed that CRS increased locomotor activity that was not ameliorated by running. It has been shown that CRS, and other chronic stress models, increase locomotor activity (Marin, Cruz and Planeta 2007; Sequeira‐Cordero et al. 2019; Shoji and Miyakawa 2020). It has been suggested that the type, intensity and number of expositions to the stressor may determine the effects on locomotor activity (Marin, Cruz and Planeta 2007; Sequeira‐Cordero et al. 2019). Under control conditions, there is a negative correlation between corticosterone levels and locomotor activity; however, after CRS this correlation is lost (Marin, Cruz and Planeta 2007). Exercise may lie in reducing some, but not all, of the health effects of stress and stress hormones. Thus, running may mitigate the altered avoidance behaviour induced by CRS but does not impact locomotor activity which may be regulated by a different neuronal circuit and neurotransmitter system, such as the dopaminergic system (Piazza et al. 1996).

We tested the role of 5‐HT_1A_ and 5‐HT_3_ receptors in regulating the excitability of vDG GCs. The modulation of GC excitability by 5‐HT is particularly relevant as GC inhibition is a key factor for stress resilience, while their hyperactivity can lead to stress‐related disorders (Schoenfeld et al. 2016; Anacker et al. 2018; Bickle et al. 2024). Previous studies have related the critical role of 5‐HT_1A_ receptors, expressed in GCs, to antidepressant responses (Samuels et al. 2015; Bickle et al. 2024), while the 5‐HT_3_ receptors have been associated with exercise‐induced antidepressant‐like phenotype (Kondo et al. 2015). Both receptors are highly expressed in the vDG (Tanaka, Samuels and Hen 2012; Dale et al. 2018). The 5‐HT_1A_ receptors are expressed in GCs and parvalbumin (PV) interneurons whereas 5‐HT_3_ receptors are expressed in cholecystokinin (CCK) interneurons (Piguet and Galvan 1994; Morales and Bloom 1997; Aznar et al. 2003). The activation of 5‐HT_1A_ receptors, a metabotropic receptor coupled to Gα_i/o_ proteins, leads to membrane hyperpolarization through the opening of inwardly rectifying potassium (GIRK) channels, resulting in decreased excitability (Albert and Vahid‐Ansari 2019), while activation of 5‐HT_3_ receptors, an ionotropic receptor permeable to cations, depolarizes CCK interneurons, leading to increased inhibitory drive onto GCs (Kawa 1994).

We found that under basal conditions, the membrane properties of the GCs remain unchanged regardless of treatment condition. However, bath application of 5‐HT revealed a differential serotonergic modulation of vDG GCs. It is possible that under non‐stressful conditions, 5‐HT release and the consequent 5‐HT receptor activation may not occur. However, when a subject faces a stressful situation, 5‐HT is released, as has been observed in the hippocampus (Vahabzadeh and Fillenz 1994), inducing activation of the 5‐HT receptors to modulate the excitability of the GCs and confer resilience (Bickle et al. 2024).

Our results show that in non‐stressed control mice, 5‐HT reduced the excitability of GCs mainly through the activation of 5‐HT_1A_ receptors, as has been observed in previous studies (Piguet and Galvan 1994). Furthermore, 5‐HT_3_ receptor activation is also involved in reducing GC excitability, as 5‐HT_3_ receptor antagonism with TROPI blocked the 5‐HT‐induced reduction in firing rate. However, this modulation is indirect, since the activation of 5‐HT_3_ receptors in GCs would increase, instead of reduce, the excitability. There are two possible explanations for this indirect modulation. The first one is the activation of 5‐HT_3_ receptors expressed in CCK interneurons (Morales and Bloom 1997) which may result in the release of GABA and the following activation of GABA_A_ extrasynaptic receptors in GCs. GABA_A_ extrasynaptic receptors are known to play a key role in regulating GCs excitability in the DG (Coulter and Carlson 2007). Indeed, 20 μM of picrotoxin was unable to block the serotonergic modulation of the firing rate as the GABA_A_ extrasynaptic receptors require high concentrations of picrotoxin to be blocked (Wlodarczyk et al. 2013). The other possibility is the activation of 5‐HT_3_ receptors expressed in adult‐born neurons that in turn inhibit GCs through GABAergic interneurons (Temprana et al. 2015; Drew et al. 2016). However, 5‐HT_3_ receptors are only expressed in stem cells and doublecortin‐expressing immature neurons (Olivas‐Cano et al. 2023).

Exposure to CRS elicited changes in stress coping, avoidance behaviours and serotonergic modulation. Electrophysiological recordings showed that the 5‐HT_1A_ receptor activation was reduced, which is consistent with their reduced activity and expression after chronic stress and corticosterone treatment in human and animal models (Neumaier et al. 2000; Savitz, Lucki and Drevets 2009; Jovanovic et al. 2011). The precise mechanism underlying this effect is unclear. Here we show that 5‐HT_1A_ receptor activation did not hyperpolarize the RMP and reduce the excitability of the GCs, as was observed in non‐stressed control mice. However, its activation reduced the R_in_ and increased the rheobase similar to non‐stressed control mice. These altered 5‐HT_1A_ receptor responses were similar to those observed when GIRK channels were ablated in ventral CA3 PYR/DG GCs and GABA_B_ receptors were activated (Vo et al. 2021). Importantly, GIRK channel ablation in these cells precluded the reduction of the avoidance behaviour induced by ML297 (a selective activator of GIRK channels) suggesting that GIRK channels are required to induce anxiolytic effects (Vo et al. 2021). It is important to highlight that GABA_B_ receptors share with 5‐HT_1A_ receptors the same G protein‐coupled GIRK channel mechanism to induce the hyperpolarizing response (Andrade, Malenka and Nicoll 1986; Andrade and Nicoll 1987) suggesting that GIRK channels may mediate, in part, the 5‐HT_1A_ receptors alterations. In GCs, three subunits of GIRK channels (GIRK1, GIRK2 and GIRK3) are highly expressed which are co‐expressed with the 5‐HT_1A_ receptor (Saenz del Burgo et al. 2008). It has been suggested that in GCs, GIRK1 and GIRK2 subunits contribute to the regulation of the R_in_, whereas the GIRK2 subunit contributes to the regulation of the somatic RMP (Gonzalez et al. 2018). Therefore, the 5‐HT_1A_ receptor dysfunction induced by CRS may be mediated by alterations of the GIRK2 subunit resulting in a lack of changes in RMP and excitability but not on R_in_ or rheobase.

Studies have shown that postsynaptic 5‐HT_1A_ receptors do not desensitise easily or internalise (Riad et al. 2001). However, corticosterone suppresses the transcription of 5‐HT_1A_ receptor mRNA and protein in the DG throughout mineralocorticoid and glucocorticoid receptors, (Meijer and de Kloet 1994; Zhong and Ciaranello 1995). Moreover, corticosterone also alters the levels of GIRK2 but not GIRK1 channel subunits in the DG (Muma and Beck 1999). Thus, CRS may alter de 5‐HT_1A_ receptor activation through the downregulation of 5‐HT_1A_ and the upregulation of the GIRK2 channel subunit inducing changes to the receptor‐effector coupling mechanism. Future studies are needed to elucidate the role of GIRK channels, and specifically the GIRK2 subunit, in the altered 5‐HT_1A_ response to 5‐HT induced by CRS, as well as in stress‐related disorders.

In addition to the 5‐HT_1A_ dysfunction in GCs, our results suggest that CRS induces the tonic activation of 5‐HT_1A_ receptors to indirectly modulate the R_in_ of GCs. Previous studies show that stress restraint increases the endogenous release of 5‐HT in the hippocampus (Joseph and Kennett 1983; Vahabzadeh and Fillenz 1994). Therefore, it is plausible that the tonic activation of 5‐HT_1A_ receptors may be mediated by an increase in the endogenous release of 5‐HT. The reduction of the R_in_ during the blocking of the 5‐HT_1A_ receptor by S‐WAY only suggests an indirect modulation as blocking the 5‐HT_1A_ receptor in GCs would increase, rather than reduce, R_in_ (Piguet and Galvan 1994). In the DG, PV interneurons also express 5‐HT_1A_ receptors (Aznar et al. 2003); therefore, CRS may modify the serotonergic modulation of GCs inducing the tonic inhibition of PV interneurons through 5‐HT_1A_ receptors. In addition, the tonic activation of 5‐HT_3_ receptors may be mediated by CCK interneurons, which are known to express this receptor (Morales and Bloom 1997). However, their blocking with TROPI does not explain the reduction of AP number in GCs. CCK interneurons unidirectionally inhibit PV interneurons to suppress their fast spiking activity (Armstrong and Soltesz 2012). Therefore, the inhibition of PV interneurons by the activation of 5‐HT_3_ receptors in CCK interneurons may reduce the inhibition of GCs. Thus, the blocking of 5‐HT_3_ receptors with TROPI would increase the inhibition of GCs resulting in the reduction of AP number as observed. Altogether suggests that CRS induces the endogenous release of 5‐HT that tonically activates both 5‐HT_3_ and 5‐HT_1A_ receptors leading to a reduced tonic GABAergic inhibition in GCs, as has been observed after chronic stress (Holm et al. 2011; Lee et al. 2016).

In runner non‐stressed mice, 5‐HT does not seem to modulate GCs excitability. However, the mechanism underlying this apparent lack of modulation was different from the one observed in mice exposed to CRS. Serotonergic modulation was mediated mainly through 5‐HT_3_ receptors, which are critical for exercise‐induced antidepressant effects (Kondo et al. 2015). In addition, tonic activation of 5‐HT_1A_ receptors expressed in PV interneurons may play a role (Aznar et al. 2003). However, previous studies suggest that 5‐HT_1A_ receptors do not seem to be critical for exercise‐induced anxiolytic effects (Rogers et al. 2016). Thus, the activation of 5‐HT_3_ receptors may increase the GABAergic inhibition onto GCs possibly by the increment of inhibitory synapses, as the amplitude and frequency of miniature inhibitory postsynaptic currents (mIPSCs) are increased after 30 days of running (Vivar, Peterson and van Praag 2016). However, this increased inhibition may be compensated by increased glutamatergic synaptic transmission in GCs after running conditions (Vivar, Peterson and van Praag 2016), resulting in an apparent lack of serotonergic modulation.

Interestingly, our results show that running recovered the altered serotonergic modulation of GCs induced by CRS. We propose that running ameliorates the altered behavioural responses elicited by CRS by rescuing the serotonergic modulation of the GCs´ excitability. Specifically, running rescued the 5‐HT_1A_ receptor activation and promoted GABAergic inhibition through 5‐HT_3_ receptor activation, as supported by the blocking of the 5‐HT modulation of the rheobase by PTX. Whether GIRK channels are related to the recovery of 5‐HT_1A_ activation remains to be investigated. However, it has been shown that 4 weeks of running reduces the corticosterone levels triggered by a post‐traumatic stress disorder model (Yakhkeshi et al. 2022). This reduction may revert the alterations induced by corticosterone in the 5‐HT_1A_ receptor and GIRK2 channel subunit (Meijer and de Kloet 1994; Zhong and Ciaranello 1995; Muma and Beck 1999). Future studies are needed to elucidate the role of exercise in the recovery of 5‐HT_1A_ receptor expression and activations, as well as GIRK channel regulation, specifically the GIRK2 subunit.

The exercise‐induced antidepressant effects mediated by 5‐HT_3_ receptors have been previously described (Kondo et al. 2015). Here we show that the running‐induced recovery of GCs serotonergic modulation of GCs excitability is also mediated by GABAergic inhibition through 5‐HT_3_ receptor activation. The CCK interneurons may be the mediators of this GABAergic inhibition as they express the 5‐HT_3_ receptors, but not the GCs (Morales and Bloom 1997). The activation of 5‐HT_3_ may result in the release of GABA and the following activation of GABA_A_ synaptic receptors, as supported by the blockade of 5‐HT modulation of rheobase by PTX (20 μM). Additionally, GABA_A_ extrasynaptic receptors in GCs may be also activated as running increases the mIPSCs, which is known to play a key role in regulating GCs´ excitability (Vivar, Peterson and van Praag 2016; Coulter and Carlson 2007). This hypothesis is supported by studies showing that running induces anxiolytic effects by engaging local inhibitory mechanisms in the ventral hippocampus (Schoenfeld et al. 2013). Thus, running may confer stress resilience through direct 5‐HT_1A_ and indirect 5‐HT_3_ receptor activation.

A limitation of this study is the absence of further *in vivo* measures of 5‐HT levels and the blocking of these receptors during behavioural testing to better understand their precise contribution to exercise‐induced resilience. However, our study provides evidence of the important role of 5‐HT, 5‐HT_1A_ and 5‐HT_3_ receptors in modulating the excitability of GCs, revelling their relevance for confers stress resilience (Bickle et al. 2024) and shedding light on potential targets for the treatment of stress‐related psychiatric disorders.

## 5 Conclusion

Our study shows that the serotonergic modulation of GC excitability may be a key factor regulating exercise‐induced stress resilience. The mechanism underlying this response is likely mediated by 5‐HT_1A_ and 5‐HT_3_ receptors that modulate directly or indirectly GC excitability. Moreover, the indirect serotonergic modulation of excitability suggests that 5‐HT may regulate GABAergic transmission through these receptors. Thus, 5‐HT_1A_ and 5‐HT_3_ receptors may be potential targets for the treatment of stress‐related psychiatric disorders.

## Author Contributions

**Carmen Soto:** conceptualization, data curation, formal analysis, funding acquisition, investigation, writing – review and editing. **Lazaro P. Orihuela:** formal analysis, funding acquisition, investigation, writing – review and editing. **Grego Apostol:** funding acquisition, investigation, writing – review and editing. **Carmen Vivar:** conceptualization, formal analysis, funding acquisition, resources, supervision, writing – original draft.

## Conflicts of Interest

The authors declare no conflicts of interests.

### Peer Review

The peer review history for this article is available at https://www.webofscience.com/api/gateway/wos/peer‐review/10.1111/ejn.70084.

## Supporting information


**TABLE S1** Two‐way ANOVA for comparisons in Figure 4.
**TABLE S2**. Two‐way ANOVA for comparisons in Figure 5.
**TABLE S3**. Paired *t*‐test 2‐tailed comparison for Figure 6. Independent experiments. 5‐HT: ACSF vs. 5‐HT; S‐WAY + 5‐HT: S‐WAY vs. S‐WAY + 5‐HT; TROPI + 5‐HT: TROPI vs. TROPI + 5‐HT.
**TABLE S4**. Paired *t*‐test 2‐tailed comparison for Figure 7. Independent experiments. S‐WAY: ACSF vs. S‐WAY; TROPI: ACSF vs. TROPI.

## Data Availability

Data are freely available upon request to the author.
