# Supplementary material for: Running Reverses Chronic Stress‐Induced Changes in Serotonergic Modulation of Hippocampal Granule Cells and Altered Behavioural Responses
Source: Eur J Neurosci. 2025 Mar 31;61(7):e70084. doi: 10.1111/ejn.70084 (PMC11959172; doi:10.1111/ejn.70084)
Supplement: Supplementary file 1 — TABLE S1 Two‐way ANOVA for comparisons in Figure 4. TABLE S2. Two‐way ANOVA for comparisons in Figure 5. TABLE S3. Paired t‐test 2‐tailed comparison for Figure 6. Independent experiments. 5‐HT: ACSF vs. 5‐HT; S‐WAY + 5‐HT: S‐WAY vs. S‐WAY + 5‐HT; TROPI + 5‐HT: TROPI vs. TROPI + 5‐HT. TABLE S4. Paired t‐test 2‐tailed comparison for Figure 7. Independent experiments. S‐WAY: ACSF vs. S‐WAY; TROPI: ACSF vs. TROPI. [file EJN-61-0-s001.pdf]

Supplementary Table 1

Two-way ANOVA for comparisons in FIGURE 4.

| Fig. | Property | Main effect | $F$ (DFn, DFd) | $p$ value |
|---|---|---|---|---|
| 4b | RMP (mV) | Stress | $F_{(1, 102)} = 1.331$ | $p = 0.2514$ |
| | | Treatment | $F_{(1, 102)} = 1.514$ | $p = 0.2214$ |
| 4c | $R_{in}$ (mW) | Stress | $F_{(1, 102)} = 0.3105$ | $p = 0.5786$ |
| | | Treatment | $F_{(1, 102)} = 0.3196$ | $p = 0.5731$ |
| 4d | Rheobase (pA) | Stress | $F_{(1, 102)} = 3.545$ | $p = 0.0626$ |
| | | Treatment | $F_{(1, 102)} = 0.05221$ | $p = 0.8197$ |

Supplementary Table 2

Two-way ANOVA for comparisons in FIGURE 5.

| Fig. | Property | Comparison | *Post hoc* Tukey´s test |
|---|---|---|---|
| 5c | RMP (mV) | -CRS+CON | ACSF *vs* +5-HT, $p = 0.0106$ |
| | | | ACSF *vs* 5-HT+PTX, $p = 0.0248$ |
| | | | 5-HT *vs* 5-HT+PTX, $p = 0.9198$ |
| | | +CRS+CON | ACSF *vs* 5-HT, $p = 0.8994$ |
| | | | ACSF *vs* 5-HT+PTX, $p = 0.8789$ |
| | | | 5-HT *vs* 5-HT+PTX, $p = 0.9989$ |
| | | -CRS+RUN | ACSF *vs* 5-HT, $p = 0.0020$ |
| | | | ACSF *vs* 5-HT+PTX, $p = 0.0002$ |
| | | | 5-HT *vs* 5-HT+PTX, $p = 0.4513$ |
| | | +CRS+RUN | ACSF *vs* 5-HT, $p = 0.0010$ |
| | | | ACSF *vs* 5-HT+PTX, $p = 0.0015$ |
| | | | 5-HT *vs* 5-HT+PTX, $p = 0.9847$ |
| 5d | $R_{in}$ (mΩ) | -CRS+CON | ACSF *vs* 5-HT, $p = 0.0007$ |
| | | | ACSF *vs* 5-HT+PTX, $p = 0.0005$ |
| | | | 5-HT *vs* 5-HT+PTX, $p = 0.9916$ |
| | | +CRS+CON | ACSF *vs* 5-HT, $p < 0.0001$ |
| | | | ACSF *vs* 5-HT+PTX, $p < 0.0001$ |
| | | | 5-HT *vs* 5-HT+PTX, $p = 0.7974$ |
| | | -CRS+RUN | ACSF *vs* 5-HT, $p = 0.0039$ |
| | | | ACSF *vs* 5-HT+PTX, $p = 0.0005$ |
| | | | 5-HT *vs* 5-HT+PTX, $p = 0.5871$ |
| | | +CRS+RUN | ACSF *vs* 5-HT, $p < 0.0001$ |
| | | | ACSF *vs* 5-HT+PTX, $p < 0.0001$ |
| | | | 5-HT *vs* 5-HT+PTX, $p = 0.5500$ |

| | | | |
|---|---|---|---|
| 5e | Rheobase (pA) | -CRS+CON | ACSF *vs* 5-HT, $p = 0.0078$<br>ACSF *vs* 5-HT+PTX, $p = 0.0003$<br>5-HT *vs* 5-HT+PTX, $p = 0.3343$ |
| | | +CRS+CON | ACSF *vs* 5-HT, $p = 0.0033$<br>ACSF *vs* 5-HT+PTX, $p = 0.0375$<br>5-HT *vs* 5-HT+PTX, $p = 0.5543$ |
| | | -CRS+RUN | ACSF *vs* 5-HT, $p = 0.1597$<br>ACSF *vs* 5-HT+PTX, $p = 0.0674$<br>5-HT *vs* 5-HT+PTX, $p = 0.8793$ |
| | | +CRS+RUN | ACSF *vs* 5-HT, $p < 0.0001$<br>ACSF *vs* 5-HT+PTX, $p = 0.0102$<br>5-HT *vs* 5-HT+PTX, $p = 0.0108$ |

Supplementary Table 3

Paired *t*-test 2-tailed comparison for FIGURE 6.
Independent experiments. 5-HT: ACSF *vs* 5-HT; S-WAY+5-HT: S-WAY *vs* S-WAY+5-HT; TROPI+5-HT: TROPI *vs* TROPI + 5-HT

| Fig. | Property | Group | Comparison | *t*-test |
|---|---|---|---|---|
| 6a | RMP % Control | -CRS+CON | 5-HT | $t_{(11)} = 4.082$, $p = 0.0018$ |
| | | | S-WAY+5-HT | $t_{(8)} = 0.01679$, $p = 0.9870$ |
| | | | TROPI+5-HT | $t_{(6)} = 3.283$, $p = 0.0168$ |
| | | +CRS+CON | 5-HT | $t_{(12)} = 0.4886$, $p = 0.6340$ |
| | | | S-WAY+5-HT | $t_{(5)} = 0.8287$, $p = 0.4450$ |
| | | | TROPI+5-HT | $t_{(5)} = 2.520$, $p = 0.0532$ |
| | | -CRS+RUN | 5-HT | $t_{(8)} = 4.005$, $p = 0.0039$ |
| | | | S-WAY+5-HT | $t_{(6)} = 2.460$, $p = 0.0491$ |
| | | | TROPI+5-HT | $t_{(7)} = 0.0891$, $p = 0.9315$ |
| | | +CRS+RUN | 5-HT | $t_{(12)} = 3.731$, $p = 0.0029$ |
| | | | S-WAY+5-HT | $t_{(6)} = 1.395$, $p = 0.2126$ |
| | | | TROPI+5-HT | $t_{(4)} = 3.193$, $p = 0.0331$ |
| 6c | $R_{in}$ % Control | -CRS+CON | 5-HT | $t_{(11)} = 6.021$, $p < 0.0001$ |
| | | | S-WAY+5-HT | $t_{(8)} = 2.150$, $p = 0.0638$ |
| | | | TROPI+5-HT | $t_{(6)} = 5.675$, $p = 0.0013$ |
| | | +CRS+CON | 5-HT | $t_{(12)} = 6.400$, $p < 0.0001$ |
| | | | S-WAY+5-HT | $t_{(5)} = 2.679$, $p = 0.0439$ |
| | | | TROPI+5-HT | $t_{(5)} = 6.235$, $p = 0.0016$ |
| | | -CRS+RUN | 5-HT | $t_{(8)} = 4.391$, $p = 0.0023$ |
| | | | S-WAY+5-HT | $t_{(6)} = 5.649$, $p = 0.0013$ |
| | | | TROPI+5-HT | $t_{(7)} = 1.410$, $p = 0.2014$ |
| | | +CRS+RUN | 5-HT | $t_{(12)} = 7.254$, $p < 0.0001$ |
| | | | S-WAY+5-HT | $t_{(6)} = 2.375$, $p = 0.0551$ |
| | | | TROPI+5-HT | $t_{(4)} = 4.117$, $p = 0.0146$ |
| 6d | Rheobase % Control | -CRS+CON | 5-HT | $t_{(11)} = 2.957$, $p = 0.0130$ |
| | | | S-WAY+5-HT | $t_{(8)} = 0.7598$, $p = 0.4692$ |
| | | | TROPI+5-HT | $t_{(6)} = 3.552$, $p = 0.0120$ |
| | | +CRS+CON | 5-HT | $t_{(12)} = 3.191$, $p = 0.0078$ |
| | | | S-WAY+5-HT | $t_{(5)} = 1.064$, $p = 0.3362$ |

| | | | | |
|---|---|---|---|---|
| | | | TROPI+5-HT | $t_{(5)} = 4.876$, $p = 0.0046$ |
| | | -CRS+RUN | 5-HT | $t_{(8)} = 2.038$, $p = 0.0759$ |
| | | | S-WAY+5-HT | $t_{(6)} = 1.894$, $p = 0.1167$ |
| | | | TROPI+5-HT | $t_{(7)} = 1.627$, $p = 0.1477$ |
| | | +CRS+RUN | 5-HT | $t_{(12)} = 6.133$, $p < 0.0001$ |
| | | | S-WAY+5-HT | $t_{(6)} = 1.295$, $p = 0.2520$ |
| | | | TROPI+5-HT | $t_{(4)} = 6.115$, $p = 0.0036$ |
| 6e | AP number % Control | -CRS+CON | 5-HT | $t_{(12)} = 0.1954$, $p = 0.8483$ |
| | | | S-WAY+5-HT | $t_{(8)} = 2.718$, $p = 0.0263$ |
| | | | TROPI+5-HT | $t_{(6)} = 0.0355$, $p = 0.9728$ |
| | | +CRS+CON | 5-HT | $t_{(11)} = 0.1512$, $p = 0.8825$ |
| | | | S-WAY+5-HT | $t_{(5)} = 0.9711$, $p = 0.3761$ |
| | | | TROPI+5-HT | $t_{(5)} = 0.4249$, $p = 0.6885$ |
| | | -CRS+RUN | 5-HT | $t_{(11)} = 0.7978$, $p = 0.4419$ |
| | | | S-WAY+5-HT | $t_{(6)} = 2.041$, $p = 0.0873$ |
| | | | TROPI+5-HT | $t_{(7)} = 0.9303$, $p = 0.3832$ |
| | | +CRS+RUN | 5-HT | $t_{(11)} = 2.569$, $p = 0.0261$ |
| | | | S-WAY+5-HT | $t_{(7)} = 0.2178$, $p = 0.8338$ |
| | | | TROPI+5-HT | $t_{(4)} = 1.854$, $p = 0.1374$ |
| 6f | Firing rate % Control | -CRS+CON | 5-HT | $t_{(12)} = 2.965$, $p = 0.0118$ |
| | | | S-WAY+5-HT | $t_{(8)} = 0.1823$, $p = 0.8599$ |
| | | | TROPI+5-HT | $t_{(6)} = 0.5971$, $p = 0.5723$ |
| | | +CRS+CON | 5-HT | $t_{(11)} = 1.856$, $p = 0.0904$ |
| | | | S-WAY+5-HT | $t_{(6)} = 0.7356$, $p = 0.4897$ |
| | | | TROPI+5-HT | $t_{(5)} = 0.7786$, $p = 0.4714$ |
| | | -CRS+RUN | 5-HT | $t_{(11)} = 0.8920$, $p = 0.3915$ |
| | | | S-WAY+5-HT | $t_{(6)} = 0.5272$, $p = 0.6170$ |
| | | | TROPI+5-HT | $t_{(7)} = 0.2720$, $p = 0.7935$ |
| | | +CRS+RUN | 5-HT | $t_{(11)} = 3.321$, $p = 0.0068$ |
| | | | S-WAY+5-HT | $t_{(7)} = 0.9737$, $p = 0.3626$ |
| | | | TROPI+5-HT | $t_{(4)} = 0.0771$, $p = 0.9422$ |

Cell number x group in RMP, $R_{in}$, and Rheobase:

5-HT:  -CRS+CON, *n* = 12 from 8 mice; +CRS+CON, *n* = 13 from 5 mice; -CRS+RUN, *n* = 9 from 6 mice; +CRS+RUN, *n* = 13 from 8 mice.

S-WAY+5-HT:  -CRS+CON, *n* = 9 from 4 mice; +CRS+CON, *n* = 7 from 4 mice; -CRS+RUN, *n* = 7 from 4 mice; +CRS+RUN, *n* = 7 from 5 mice.

TROPI+5-HT:  -CRS+CON, *n* = 7 from 4 mice; +CRS+CON, *n* = 6 from 4 mice; -CRS+RUN, *n* = 8 from 4 mice; +CRS+RUN, *n* = 5 from 4 mice.

Cell number x group in AP number and Firing rate

5-HT:  -CRS+CON, *n* = 13 from 10 mice; +CRS+CON, *n* = 12 from 5 mice; -CRS+RUN, *n* = 12 from 10 mice; +CRS+RUN, *n* = 12 from 7 mice.

S-WAY+5-HT:  -CRS+CON, *n* = 9 from 4 mice; +CRS+CON, *n* = 7 from 4 mice; -CRS+RUN, *n* = 7 from 4 mice; +CRS+RUN, *n* = 8 from 5 mice.

TROPI+5-HT:  -CRS+CON, *n* = 7 from 4 mice; +CRS+CON, *n* = 6 from 3 mice; -CRS+RUN, *n* = 8 from 4 mice; +CRS+RUN, *n* = 5 from 3 mice.

Supplementary Table 4

Paired *t*-test 2-tailed comparison for FIGURE 7.

Independent experiments. S-WAY: ACSF *vs* S-WAY; TROPI: ACSF *vs* TROPI.

| Fig. | Property | Group | Comparison | *t*-test |
|---|---|---|---|---|
| 7a | RMP % Control | -CRS+CON | S-WAY | $t_{(8)} = 0.5077$, $p = 0.6254$ |
| | | | TROPI | $t_{(6)} = 0.6755$, $p = 0.5245$ |
| | | +CRS+CON | S-WAY | $t_{(5)} = 0.8925$, $p = 0.4130$ |
| | | | TROPI | $t_{(5)} = 1.583$, $p = 0.1743$ |
| | | -CRS+RUN | S-WAY | $t_{(6)} = 3.398$, $p = 0.0145$ |
| | | | TROPI | $t_{(7)} = 0.0614$, $p = 0.9527$ |
| | | +CRS+RUN | S-WAY | $t_{(6)} = 0.9134$, $p = 0.3963$ |
| | | | TROPI | $t_{(4)} = 1.193$, $p = 0.2987$ |
| 7b | $R_{in}$ % Control | -CRS+CON | S-WAY | $t_{(8)} = 0.4148$, $p = 0.6892$ |
| | | | TROPI | $t_{(6)} = 0.1008$, $p = 0.9230$ |
| | | +CRS+CON | S-WAY | $t_{(5)} = 2.693$, $p = 0.0431$ |
| | | | TROPI | $t_{(5)} = 1.369$, $p = 0.2293$ |
| | | -CRS+RUN | S-WAY | $t_{(6)} = 1.369$, $p = 0.2202$ |
| | | | TROPI | $t_{(7)} = 1.922$, $p = 0.0961$ |
| | | +CRS+RUN | S-WAY | $t_{(6)} = 0.0111$, $p = 0.9914$ |
| | | | TROPI | $t_{(4)} = 0.6531$, $p = 0.5493$ |
| 7c | Rheobase % Control | -CRS+CON | S-WAY | $t_{(8)} = 0.3416$, $p = 0.7414$ |
| | | | TROPI | $t_{(6)} = 0.4579$, $p = 0.6631$ |
| | | +CRS+CON | S-WAY | $t_{(5)} = 1.265$, $p = 0.2617$ |
| | | | TROPI | $t_{(5)} = 0.7262$, $p = 0.5003$ |
| | | -CRS+RUN | S-WAY | $t_{(6)} = 0.4224$, $p = 0.6875$ |
| | | | TROPI | $t_{(7)} = 0.3693$, $p = 0.3693$ |
| | | +CRS+RUN | S-WAY | $t_{(6)} = 1.365$, $p = 0.2213$ |
| | | | TROPI | $t_{(4)} = 0.5213$, $p = 0.6297$ |

| | | | | |
|---|---|---|---|---|
| 7d | AP number % Control | -CRS+CON | S-WAY | $t_{(8)} = 0.5380$, $p = 0.6052$ |
| | | | TROPI | $t_{(6)} = 1.388$, $p = 0.2144$ |
| | | +CRS+CON | S-WAY | $t_{(5)} = 0.2921$, $p = 0.7820$ |
| | | | TROPI | $t_{(5)} = 2.767$, $p = 0.0395$ |
| | | -CRS+RUN | S-WAY | $t_{(6)} = 2.532$, $p = 0.0446$ |
| | | | TROPI | $t_{(7)} = 0.8950$, $p = 0.4005$ |
| | | +CRS+RUN | S-WAY | $t_{(6)} = 1.432$, $p = 0.2021$ |
| | | | TROPI | $t_{(4)} = 1.961$, $p = 0.1214$ |
| 7e | Firing rate % Control | -CRS+CON | S-WAY | $t_{(8)} = 0.5070$, $p = 0.6258$ |
| | | | TROPI | $t_{(6)} = 0.3815$, $p = 0.7160$ |
| | | +CRS+CON | S-WAY | $t_{(5)} = 0.9816$, $p = 0.3714$ |
| | | | TROPI | $t_{(5)} = 0.5571$, $p = 0.6015$ |
| | | -CRS+RUN | S-WAY | $t_{(6)} = 1.038$, $p = 0.3391$ |
| | | | TROPI | $t_{(7)} = 0.3802$, $p = 0.7151$ |
| | | +CRS+RUN | S-WAY | $t_{(6)} = 1.031$, $p = 0.3422$ |
| | | | TROPI | $t_{(4)} = 0.2510$, $p = 0.8142$ |

Cell number x group

S-WAY+5-HT: -CRS+CON, $n = 9$ from 4 mice; +CRS+CON, $n = 6$ from 4 mice; -CRS+RUN, $n = 7$ from 4 mice; +CRS+RUN, $n = 7$ from 5 mice.

TROPI+5-HT: -CRS+CON, $n = 7$ from 4 mice; +CRS+CON, $n = 6$ from 3 mice; -CRS+RUN, $n = 8$ from 4 mice; +CRS+RUN, $n = 5$ from 3 mice.
